# ESX-1-Independent Horizontal Gene Transfer by Mycobacterium tuberculosis Complex Strains

**DOI:** 10.1128/mBio.00965-21

**Published:** 2021-05-18

**Authors:** Jan Madacki, Mickael Orgeur, Guillem Mas Fiol, Wafa Frigui, Laurence Ma, Roland Brosch

**Affiliations:** a Institut Pasteur, Unit for Integrated Mycobacterial Pathogenomics, CNRS UMR 3525, Paris, France; b Institut Pasteur, C2RT, Biomics, Paris, France; University of Massachusetts—Amherst

**Keywords:** DNA transfer, ESX-1, *Mycobacterium canettii*, *Mycobacterium tuberculosis*, conjugation, recombinant

## Abstract

Current models of horizontal gene transfer (HGT) in mycobacteria are based on “distributive conjugal transfer” (DCT), an HGT type described in the fast-growing, saprophytic model organism Mycobacterium smegmatis, which creates genome mosaicism in resulting strains and depends on an ESX-1 type VII secretion system. In contrast, only few data on interstrain DNA transfer are available for tuberculosis-causing mycobacteria, for which chromosomal DNA transfer between two Mycobacterium canettii strains was reported, a process which, however, was not observed for Mycobacterium tuberculosis strains. Here, we have studied a wide range of human- and animal-adapted members of the Mycobacterium tuberculosis complex (MTBC) using an optimized filter-based mating assay together with three selected strains of M. canettii that acted as DNA recipients. Unlike in previous approaches, we obtained a high yield of thousands of recombinants containing transferred chromosomal DNA fragments from various MTBC donor strains, as confirmed by whole-genome sequence analysis of 38 randomly selected clones. While the genome organizations of the obtained recombinants showed mosaicisms of donor DNA fragments randomly integrated into a recipient genome backbone, reminiscent of those described as being the result of ESX-1-mediated DCT in M. smegmatis, we observed similar transfer efficiencies when ESX-1-deficient donor and/or recipient mutants were used, arguing that in tubercle bacilli, HGT is an ESX-1-independent process. These findings provide new insights into the genetic events driving the pathoevolution of M. tuberculosis and radically change our perception of HGT in mycobacteria, particularly for those species that show recombinogenic population structures despite the natural absence of ESX-1 secretion systems.

## INTRODUCTION

Horizontal gene transfer (HGT) is a major factor in bacterial evolution, and it has shaped the genomes of many important pathogens (1, 2). In mycobacteria, evidence of genetic transfer between different strains of the saprophytic bacterium Mycobacterium smegmatis started to emerge decades ago (3–5), and the research on this subject was revisited and extended years later (6). From this research, it became apparent that M. smegmatis uses a unique form of conjugal chromosomal DNA transfer whereby DNA is unidirectionally transferred from one strain (the donor) to a second strain (the recipient). This transfer probably originates from multiple transfer initiation sites, requiring recipient recombination functions as well as extended homology between the transferred donor DNA and the recipient chromosome (7, 8). This form of transfer was designated distributive conjugal transfer (DCT) because it results in mosaic transconjugant genomes containing several donor DNA segments randomly distributed around the chromosome (9).

How such DNA transfer is established across the mycobacterial cell envelope remains a challenging question. Indeed, while phylogenetically considered for a long time to be Gram-positive bacteria, mycobacteria possess a complex cell envelope that is composed of a plasma membrane, a peptidoglycan layer, an arabinogalactan layer, a highly hydrophobic outer membrane (mycomembrane), and a capsule, characterizing them functionally as diderm bacteria (10, 11). However, they seem to lack the traditional components required for HGT, and the exact mechanisms of mating pair formation and DNA transfer itself still need to be elucidated. As one important factor, the involvement of M. smegmatis type VII (ESX) secretion systems in these processes was reported. Although only components of the transport machinery across the plasma membrane have been identified so far (12–14), these secretion systems are thought to be specialized for the secretion of various protein substrates across the complex cell envelope (15, 16), with five functionally nonredundant ESX systems being present in Mycobacterium tuberculosis, the causative agent of tuberculosis, and only three being present in M. smegmatis (ESX-1, ESX-3, and ESX-4) (17). It was observed that in M. smegmatis recipient strains, ESX-1 and ESX-4 secretion systems were indispensable for conjugation, while deletions in the donor ESX-1 secretion system resulted in a higher conjugation efficiency (18–20), leading to the conclusion that these secretion systems might facilitate cell-cell communication (21, 22). Interestingly, genetic determinants that define whether an M. smegmatis strain is able to act as a donor or as a recipient strain were mapped to a six-gene mating identity (*mid*) segment in the 3′ region of the *esx-1* locus, the replacement of which was linked to a switch from a recipient phenotype to a donor phenotype (9). In tuberculosis-causing mycobacteria, the ESX-1 secretion system is known to be a key virulence determinant, with its absence causing severe attenuation, as seen in the ESX-1-deleted vaccine strains Mycobacterium bovis bacillus Calmette-Guérin (BCG) (23, 24) and Mycobacterium microti MP Prague (25).

A form of chromosomal DNA transfer highly resembling DCT was also observed between two strains of Mycobacterium canettii, the only example to date of experimentally demonstrated chromosomal DNA transfer in slow-growing mycobacteria (26). M. canettii strains bear a close resemblance to the putative progenitor of modern M. tuberculosis lineages, and they show numerous signs of interstrain recombination (27, 28). The extent to which the evolution of pathogenic mycobacteria, most notably M. tuberculosis, was shaped by HGT has been a subject of several studies, and in contrast to the rare exception of results from a polymorphism-based sequence analysis (29), it is now generally accepted that HGT was a major driving force of its evolution before an apparent evolutionary bottleneck after which the M. tuberculosis complex (MTBC) evolved by clonal expansion into various lineages of human- and animal-adapted tubercle bacilli (27, 30–36). Whereas extensive genome mosaicism was demonstrated in several *M. canettii* genomes, suggesting that HGT was widespread in the *M. canettii*-like progenitor pool from which the MTBC emerged (28, 37–39), the clonal genomic population structure in the extant MTBC suggested that no such transfer happens in and among MTBC lineages after branching from *M. canettii*. The question of whether the ability for HGT in extant M. tuberculosis is preserved remains an important point of discussion (22, 26, 40) given the strong impact of HGT on the emergence of drug resistance and virulence in many other human pathogens (2).

In this work, we have thus investigated the ability for interstrain DNA transfer in a wide range of wild-type (WT) and mutant strains of various tubercle bacilli, including representative MTBC members and *M. canettii* strains. Our results revealed that the donor capacity for the transmission of chromosomal DNA into other tubercle bacilli is indeed still active in a wide range of MTBC members and *M. canettii* strains, whereas only a few *M. canettii* strains and no MTBC strains were able to act as recipient strains in the DNA transfer experiments. To our large surprise, we also did not find any evidence of the potential involvement of ESX-1 type VII secretion systems in DNA transfer among tubercle bacilli, suggesting that mechanisms of HGT among slow-growing mycobacteria might be quite different from those reported for the fast-growing M. smegmatis model despite similar patterns of randomly distributed transferred DNA fragments in both cases.

## RESULTS

### Tuberculosis-causing mycobacteria are successful donors of chromosomal DNA.

**FIG 1:**
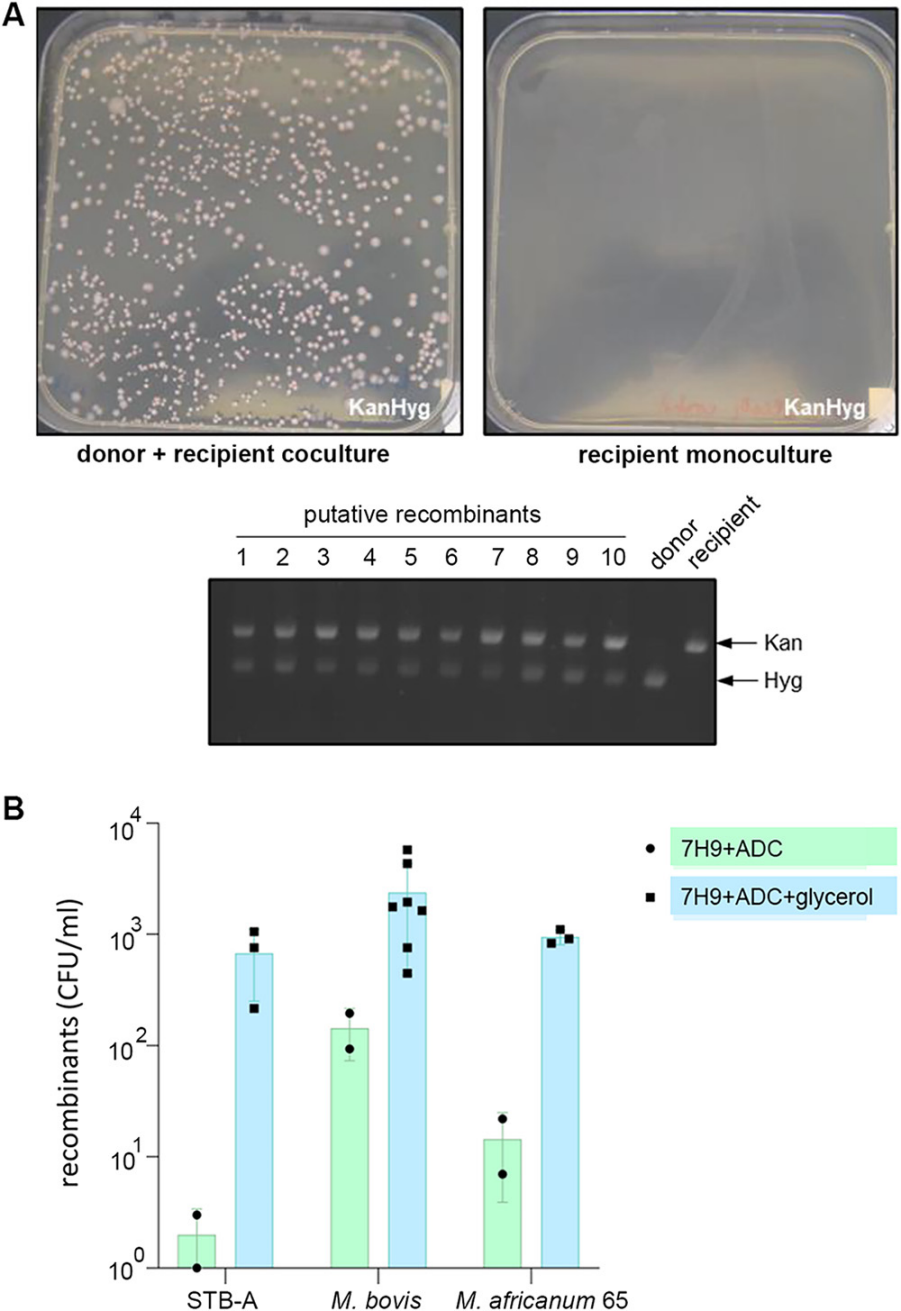
Optimized mating assay. (A) Example of results from the optimized mating assay showing a large number of double-resistant colonies obtained from a donor, carrying a hygromycin resistance cassette on integrated plasmid pYUB412, and a recipient, carrying a kanamycin resistance cassette on episomal plasmid pMRF1-dsRed, coculture as well as the absence of colonies when the monoculture of the recipient was plated onto 7H11 plates containing kanamycin and hygromycin. PCR confirmed the presence of the two antibiotic markers in 10 randomly selected recombinants as well as donor and recipient strains. (B) Number of recovered recombinants resulting from mating assays with STB-A, M. bovis AF2122/97, and M. africanum 65 donor strains and the STB-L recipient strain when cultures were grown in 7H9 medium with 10% ADC and 0.05% Tween 80, or 7H9 medium with 10% ADC, 0.05% Tween 80 and 0.2% glycerol prior to the assays. Note that M. bovis AF2122/97 and M. africanum 65 cultures were also routinely supplemented with 0.2% pyruvate. Bars represent means ± standard deviations (SD).

**FIG 2:**
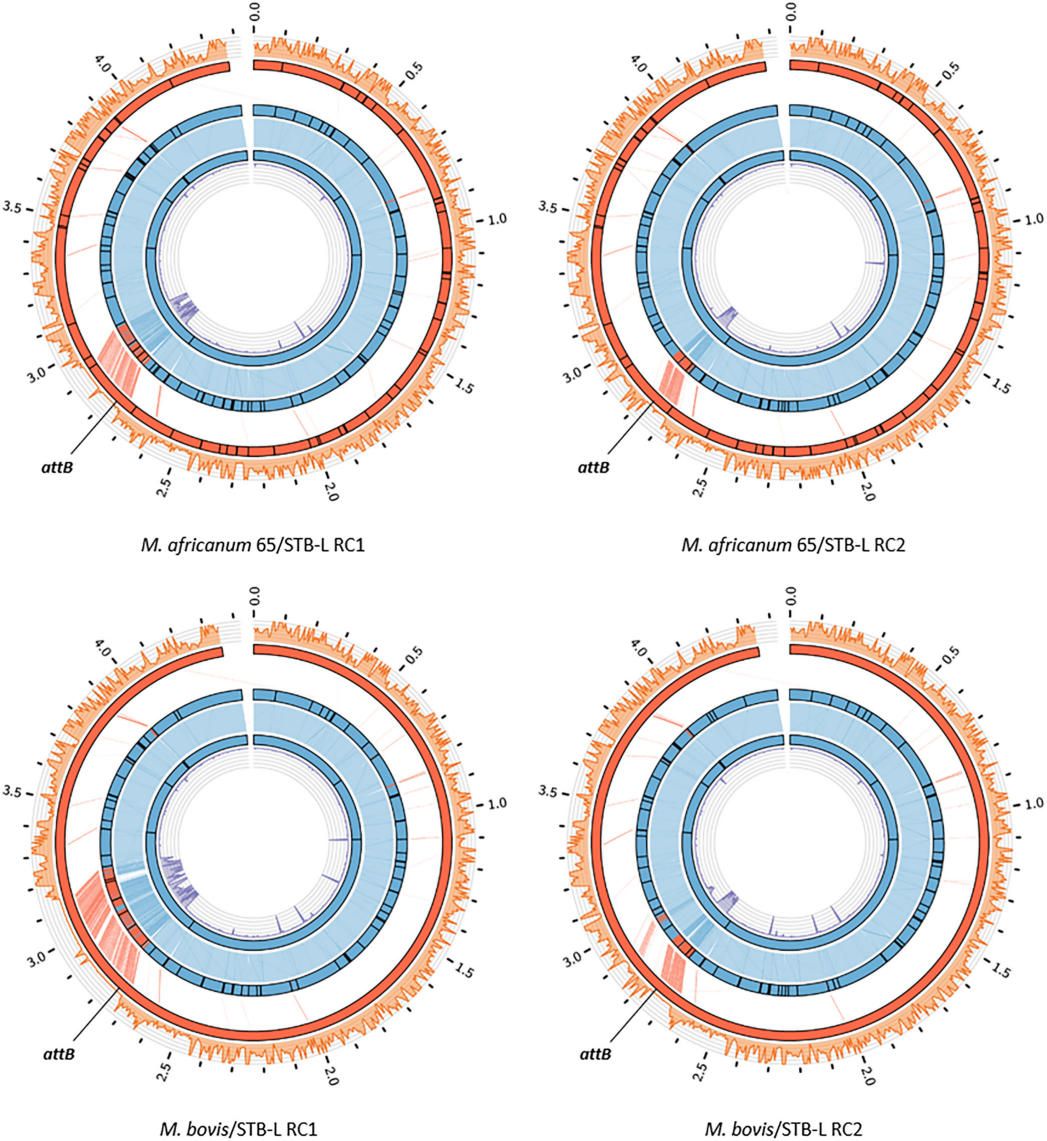
Circular representation of genomes from recombinants. The genomes of recombinant 1 (RC1) and recombinant 2 (RC2) shown in the top row were obtained when M. africanum 65 was used as the donor strain together with *M. canettii* STB-L as the recipient strain (M. africanum 65/STB-L RC1 and M. africanum 65/STB-L RC2). Similarly, the genomes of RC1 and RC2 shown in the bottom row were obtained when M. bovis AF2122/97 was used as the donor strain together with *M. canettii* STB-L as the recipient strain (M. bovis/STB-L RC1 and M. bovis/STB-L RC2). From the outer to the inner circle are (i) the density of detected variants calculated in 5-kb nonoverlapping windows between the recombinant and donor strains (orange), (ii) the donor strain reference genome (red), (iii) the best-scoring hits identified between the recombinant and donor strains (light red), (iv) the assembled recombinant genome (red, region assigned to the donor strain; blue, region assigned to the recipient strain; white, region of unknown origin), (v) the best-scoring hits identified between the recombinant and recipient strains (light blue), (vi) the recipient strain reference genome (blue), and (vii) the density of detected variants calculated in 5-kb nonoverlapping windows between the recombinant and recipient strains (purple). Black bars correspond to gap regions. Coordinates are indicated in megabases. *attB*, L5 integration site. Note that a considerable portion of the transferred sequences is usually localized in the proximity of the *attB* L5 integration site because at this site of the donor genome, vector pYUB412 is integrated, which carries an antibiotic resistance marker used for the selection of double-antibiotic-resistant recombinants.

**FIG 3:**
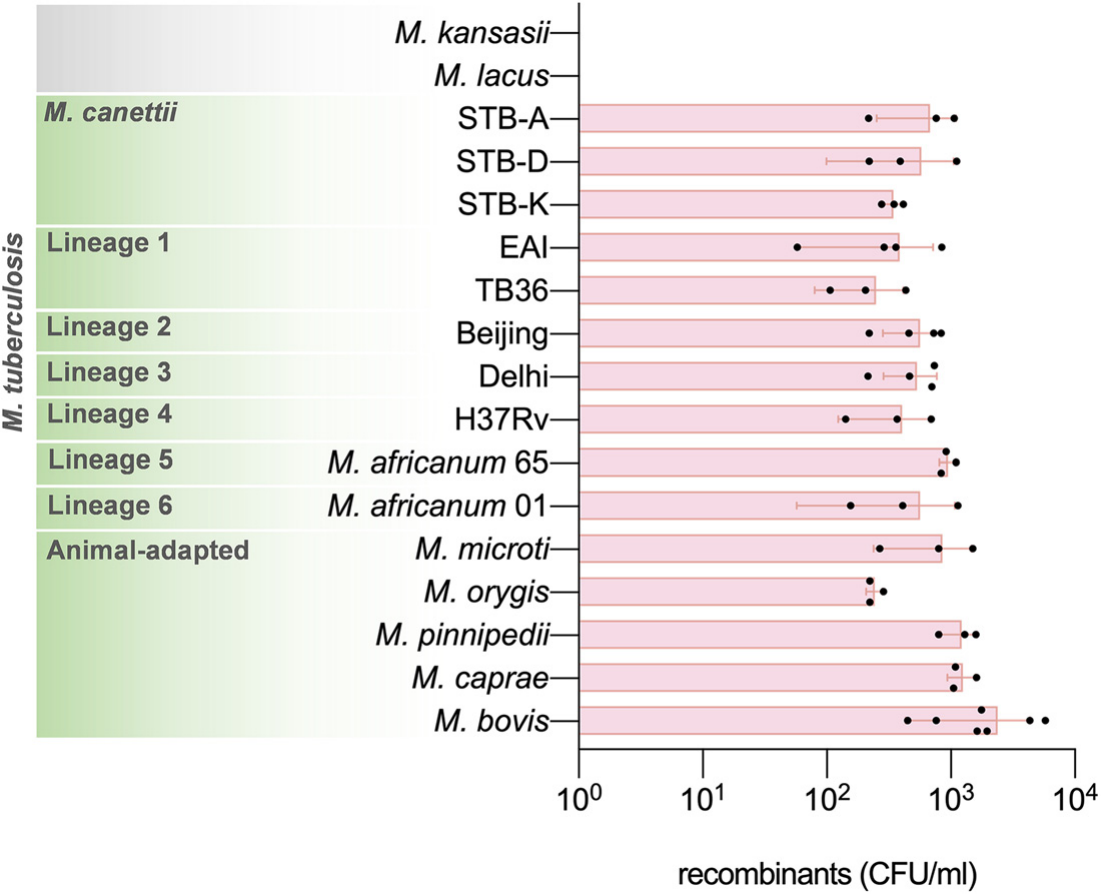
Ability of selected slow-growing mycobacteria to act as donor strains in chromosomal DNA transfer. Numbers of recovered recombinants in mating assays with STB-L as the recipient strain are shown. At least two independent mating assays were performed per mating pair. Reproducibly, no double-antibiotic-resistant colonies were recovered when plating the recipient monoculture. Spontaneous kanamycin-resistant donor colonies, if any, were distinguished by the lack of DsRed production. Bars represent means ± SD.

The originally observed transfer of several genomic DNA fragments from *M. canettii* strain A (STB-A) (CIPT 140010059) to *M. canettii* strain L (STB-L) (CIPT 140070008) (26) prompted us to investigate if such DNA transfer occurrences were also possible for other tubercle bacilli and in particular members of the MTBC. In our initial experiments, alongside the previously demonstrated donor STB-A as a control, we introduced the integrative Hyg^r^ plasmid pYUB412 (41) into strains of Mycobacterium africanum CIPT 140030065 and M. bovis AF2122/97 and tested them as potential donor strains together with the previously used *M. canettii* recipient strain STB-L, which carried a nonmobilizable replicative pMRF1-dsRed Kan^r^ plasmid. Potential mating pairs were incubated on solid medium, allowing close physical contact of bacteria, as previously described (6, 26), and DsRed-producing Hyg^r^ Kan^r^ recombinants were selected for further analysis (Fig. 1A). Interestingly, all three pairs provided double-antibiotic-resistant colonies, and randomly selected colonies were tested and confirmed for the presence of both antibiotic cassettes by PCR (Fig. 1A). We observed a higher number of these colonies when, prior to mating, strains were grown in the presence of glycerol (Fig. 1B), underlining the importance of a nutrient-rich medium for DNA transfer (42, 43). When DNA preparations from selected colonies were then subjected to Illumina-based whole-genome sequencing (WGS) and *de novo* assembly of reads, bioinformatic analysis identified various DNA stretches from M. africanum or M. bovis that were embedded within the STB-L-like genome backbone of the double-resistant recombinants (Fig. 2). In follow-up experiments, we tested a wide range of *M. canettii* and MTBC strains to evaluate if double-resistant colonies could be obtained by using STB-L as a common recipient strain. All of the 15 tested *M. canettii* and MTBC strains generated a large number of recombinants in combination with STB-L, indicating that the respective strains were able to act as donors (Fig. 3). In contrast, no such recombinants were obtained when Mycobacterium kansasii or Mycobacterium lacus control strains, representing nontuberculous mycobacteria (NTM) with different degrees of relatedness to the MTBC (44), were used as potential donor strains, suggesting that the observed interstrain recombination events among the other strains were specific to tubercle bacilli.

### Evaluation of the role of ESX-1 in chromosomal DNA transfer in slow-growing mycobacteria.

**FIG 4:**
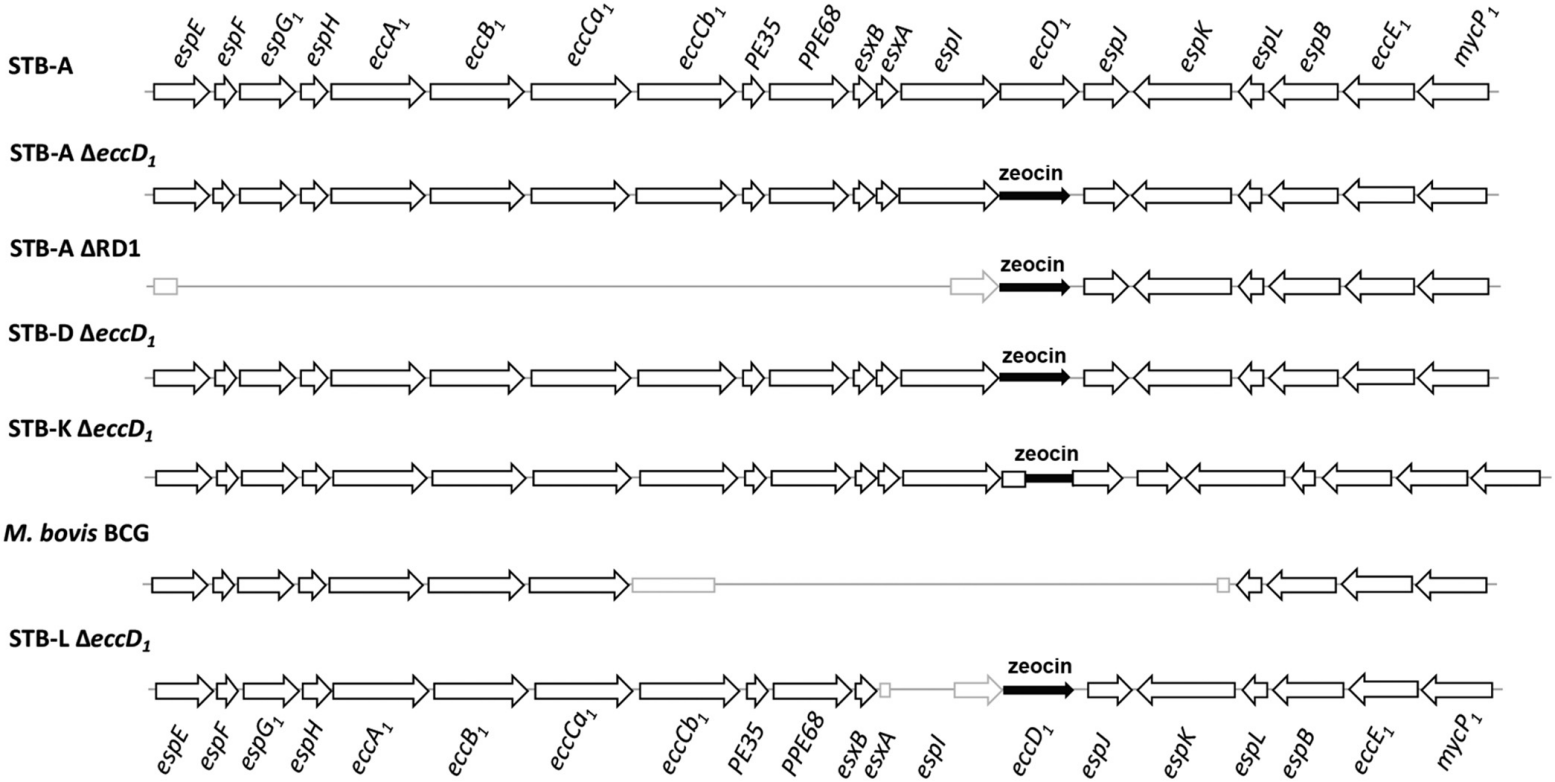
Schematic representation of the *esx-1* locus in different mutant strains used in mating assays. *M. canettii* strains STB-A Δ*eccD_1_*, STB-A ΔRD1, STB-D Δ*eccD_1_*, and STB-L Δ*eccD_1_* contain the gene conferring resistance to zeocin (represented by a full black arrow), replacing the entire coding sequence of gene *eccD_1_*. In the case of STB-A ΔRD1, there is an additional deletion between genomic coordinates 4410645 and 4424524 of the STB-A reference genome, spanning genes *espE* to *espI*. An additional deletion upstream of *eccD_1_* is also found in STB-L Δ*eccD_1_* starting from position 220 of the *esxA* open reading frame (ORF) and ending at position 1364 of the *espI* ORF. STB-K Δ*eccD_1_* has the zeocin cassette inserted between positions 1247 and 1256 of the *eccD_1_* ORF. Each of the described deletions was confirmed by WGS, which also served to confirm the absence of any other mutations in these strains, which were then used in different mating assays.

As deletions in the *esx-1* locus in M. smegmatis were reported to cause opposing effects on donor and recipient strains, whereby ESX-1 deficiency of the donor strain increased the transfer efficiency and ESX-1 deficiency in the recipient strain disabled transfer (20), we sought to evaluate if these roles in HGT were preserved in slow-growing mycobacteria. We therefore constructed mutants of *M. canettii* strains STB-A, STB-D, and STB-K, in which *eccD_1_*, a gene coding for the conserved component of the ESX-1 machinery, was replaced or disrupted with a zeocin resistance cassette (Fig. 4), and used them as donors in mating assays with STB-L. When constructing the STB-A Δ*eccD_1_* mutant, we obtained two strains with different genotypes: one with a zeocin resistance cassette replacing the open reading frame (ORF) of *eccD_1_*, as expected, and a second one showing an additional, probably spontaneous, deletion of a 13.9-kb region starting 806 bp upstream of *eccD_1_*, roughly coinciding with the RD1^mic^ region deletion found in M. microti (25) (Fig. 4). The latter strain was named STB-A ΔRD1 here and used in addition to the *eccD_1_* knockout mutants of STB-A, STB-D, and STB-K in mating experiments. Moreover, we also included the reference strain M. tuberculosis H37Rv (45), an ESX-1-deficient mutant strain named H37Rv ΔRD1 (23), and the reference strain M. bovis AF2122/97 (46) as well as three BCG substrains (Russia, Tokyo, and Pasteur) (47), which are well known to lack ESX-1 functions due to the deletion of the region of difference RD1 (23, 24).

**FIG 5:**
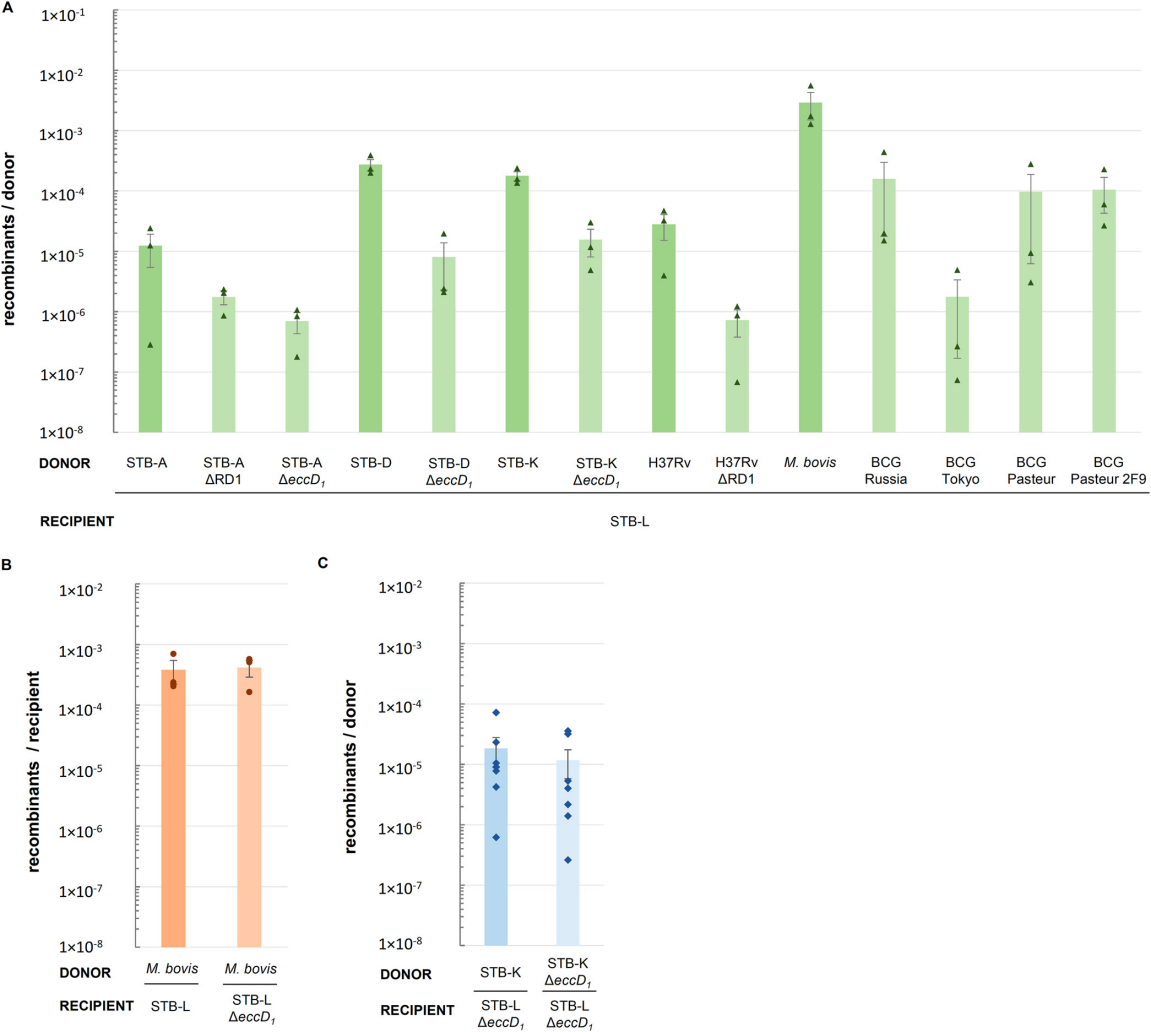
DNA transfer efficiency in different ESX-1 mutant strains and the corresponding WT strains. (A) Donor ESX-1 mutant strains with STB-L as the recipient strain. The transfer efficiency is expressed as the number of recombinants per recovered donor cell. (B) STB-L Δ*eccD_1_* recipient strain with M. bovis AF2122/97 as the donor strain. The transfer efficiency is expressed as the number of recombinants per recovered recipient cell. (C) STB-K Δ*eccD_1_* donor strain with the STB-L Δ*eccD_1_* recipient strain. The transfer efficiency is expressed as the number of recombinants per recovered donor cell. Bars represent means ± standard errors of the means (SEM). Statistical analysis was performed using a Kruskal-Wallis test followed by Dunn’s multiple-comparison test when three or more groups were compared or a Mann-Whitney test when two groups were compared.

The results of quantitative mating assays with STB-L as a recipient revealed that none of the four ESX-1-deficient *M. canettii* donor mutants generated a higher DNA transfer efficiency than the corresponding WT strains (Fig. 5A). Interestingly, the mutant strains even showed a slight reduction, albeit nonsignificant, of their ability for transfer, ranging from a 7-fold decrease in the transfer efficiency for STB-A ΔRD1 compared to STB-A WT to a 34-fold decrease for STB-D Δ*eccD_1_* compared to STB-D WT (Fig. 5A). Moreover, similar results were also obtained when the transfer efficiencies of M. tuberculosis H37Rv WT and M. tuberculosis H37Rv ΔRD1 or BCG Russia, Tokyo, and Pasteur substrains were compared (Fig. 5A). Finally, we also used a recombinant BCG Pasteur strain in which ESX-1 functions had been restored by the integration of the cosmid pRD1-2F9 containing an intact *esx-1* locus from M. tuberculosis H37Rv (24) and recorded that it provided a transfer efficiency similar to that of BCG Pasteur (Fig. 5A).

As for M. smegmatis, the presence of a functional ESX-1 system in the recipient strain was reported to be an essential requirement for transfer (20), we also sought to test the involvement of the recipient ESX-1 secretion system in the genetic background of tubercle bacilli. Thus, an *eccD_1_* mutant of the STB-L recipient strain was constructed whereby *eccD_1_* was disrupted (Fig. 4). In addition, we observed that during Δ*eccD_1_* mutant construction, an additional deletion in the *esx-1* locus, affecting the upstream genes *espI* and *esxA*, occurred (Fig. 4; see also Fig. S1 in the supplemental material), which further ensured the nonfunctionality of the ESX-1 system in this mutant, similar to the situation observed previously for *M. canettii* strain STB-A ΔRD1, as described above. When mating experiments were conducted using M. bovis AF2122/97 as the donor and STB-L Δ*eccD_1_* as the recipient, unexpectedly, we observed a transfer efficiency as high as that for control mating experiments conducted with the WT M. bovis AF2122/97 donor and STB-L recipient strains, indicating that genomic DNA transfer between tubercle bacilli does not require an ESX-1-proficient recipient strain (Fig. 5B). To check for a possible functional redundancy of donor and recipient ESX-1 systems in the process, we performed mating assays between STB-K Δ*eccD_1_* as a donor and STB-L Δ*eccD_1_* as a recipient. The resulting efficiency of DNA transfer between these two *eccD_1_*-deficient strains was comparable to the one where STB-K WT was used as the donor, further confirming the dispensability of ESX-1 systems for transfer (Fig. 5C).

10.1128/mBio.00965-21.1 FIG S1 Display of mapped Illumina sequencing reads generated from recipient strain STB-L Δ*eccD_1_*-pMRF1-dsRed and the recombinant M. bovis/STB-L Δ*eccD_1_* RC1 relative to the genome of wild-type (WT) STB-L. Note the absence of reads for gene *eccD_1_* (*BN43_90410*) in Δ*eccD_1_* strains as well as the lack of reads concerning an additional deletion affecting genes *esxA* and *espI* (*BN43_90409*). The absence of reads confirms the deletion of genes in the Δ*eccD_1_* recipient and recombinant that are essential for the function of the ESX-1 system. Download FIG S1, PDF file, 2.7 MB. Copyright © 2021 Madacki et al. 2021 Madacki et al. https://creativecommons.org/licenses/by/4.0/ This content is distributed under the terms of the Creative Commons Attribution 4.0 International license.

### Whole-genome sequencing of recombinants.

**FIG 6:**
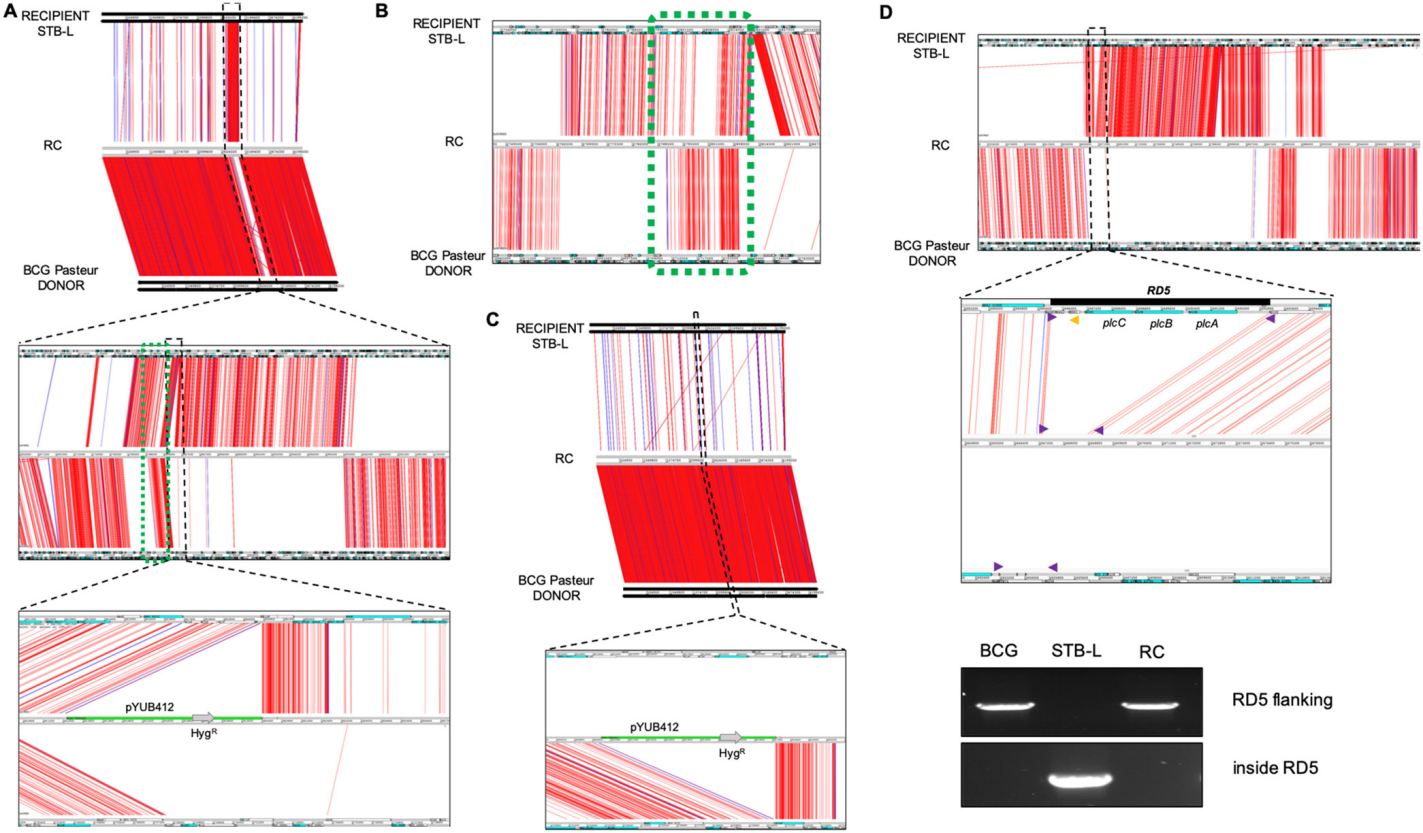
Examples of Artemis Comparison Tool (ACT) visualization of variants detected between recombinants (RC) and donor and recipient strains. SNPs that differ between genomes are represented by red and indels by blue lines. (A) BCG Pasteur/STB-L RC2, where sequences flanking the pYUB412 integration site were transferred from the donor strain. (B) Example of a region of BCG Pasteur/STB-L RC2 depicting microcomplexity (green dotted frame). (C and D) BCG Pasteur/STB-L RC1, where no apparent donor-derived segments are found flanking the pYUB412 sequence (C), and BCG Pasteur/STB-L RC3, where a chromosomal DNA transfer-related RD5 deletion had occurred (D). PCR confirmed the RD5 deletion in the recombinant (primers flanking the RD5 region are marked with purple arrows, and a primer inside the RD5 region is marked with a yellow arrow).

From the numerous double-resistant recombinants obtained from mating experiments, a representative range was selected to be subjected to WGS (Fig. S2). Recombinant genomes were *de novo* assembled from sequencing reads and compared against their corresponding donor and recipient genomes to distinguish donor-derived sequences from recipient sequences based on polymorphism signals detected between them. Bioinformatic analysis followed by visualization with the Artemis Comparison Tool (ACT) (48) revealed that for the majority of recombinants, a variably sized chromosomal segment next to the *attB* site of the hygromycin cassette-containing integrative vector was transferred, which was accompanied by several other smaller segments from randomly transferred parts of the donor genome (Fig. 2; Fig. S2 and Table S1). These strains contained 1 to 14 continuous regions of the recipient genome replaced by donor-derived chromosomal DNA with sizes ranging from 0.3 to 190.3 kb and a total size of recipient DNA exchanged for donor DNA per recombinant strain of between 15.2 and 389.1 kb. Regions of microcomplexity up to 18.6 kb, showing reduced identity compared to both donor and recipient reference sequences, were also present in some recombinants (Fig. 6A and B; Table S1), similar to those seen in M. smegmatis (9). In one recombinant clone obtained from mating BCG Pasteur and STB-L, however, there was no apparent mycobacterial donor-derived chromosomal DNA present, and we found only the sequence of the integrated pYUB412 vector containing the hygromycin resistance cassette in the genome of the recombinant, a finding that was in contrast to other recombinants of the same mating pair where different portions of BCG Pasteur-derived DNA were present in the recombinants (Fig. 6C; Fig. S2C and D and Table S1). This result is surprising and could have arisen from posttransfer recombination events and/or integrase-mediated excision/integration of vector pYUB412 (49).

10.1128/mBio.00965-21.2 FIG S2 Circular representation of 21 selected genomes from recombinants (RCs) obtained when STB-L and/or STB-L Δ*eccD_1_* was used as the recipient strain with the indicated donors. From the outer to the inner circle are (i) the density of detected variants calculated in 5-kb nonoverlapping windows between the recombinant and donor strains (orange), (ii) the donor strain reference genome (red), (iii) the best-scoring hits identified between the recombinant and donor strains (light red), (iv) the assembled recombinant genome (red, region assigned to the donor strain; blue, region assigned to the recipient strain; white, region of unknown origin), (v) the best-scoring hits identified between the recombinant and recipient strains (light blue), (vi) the recipient strain reference genome (blue), and (vii) the density of detected variants calculated in 5-kb nonoverlapping windows between the recombinant and recipient strains (purple). Black bars correspond to gap regions. Coordinates are indicated in megabases. Download FIG S2, PDF file, 2.0 MB. Copyright © 2021 Madacki et al. 2021 Madacki et al. https://creativecommons.org/licenses/by/4.0/ This content is distributed under the terms of the Creative Commons Attribution 4.0 International license.

10.1128/mBio.00965-21.6 TABLE S1 List of recombinant strains that were subjected to WGS. Donor-derived sequences were identified based on polymorphism signals between the recombinant sequence and the recipient reference sequence. For each fragment, start and end positions on the recipient reference genome are given. M, regions of microcomplexity; DS, regions where additional donor-specific sequences are present inside the transferred fragment. For each of these fragments, start and end positions on the donor reference genome are also given. Download Table S1, PDF file, 0.07 MB. Copyright © 2021 Madacki et al. 2021 Madacki et al. https://creativecommons.org/licenses/by/4.0/ This content is distributed under the terms of the Creative Commons Attribution 4.0 International license.

**FIG 7:**
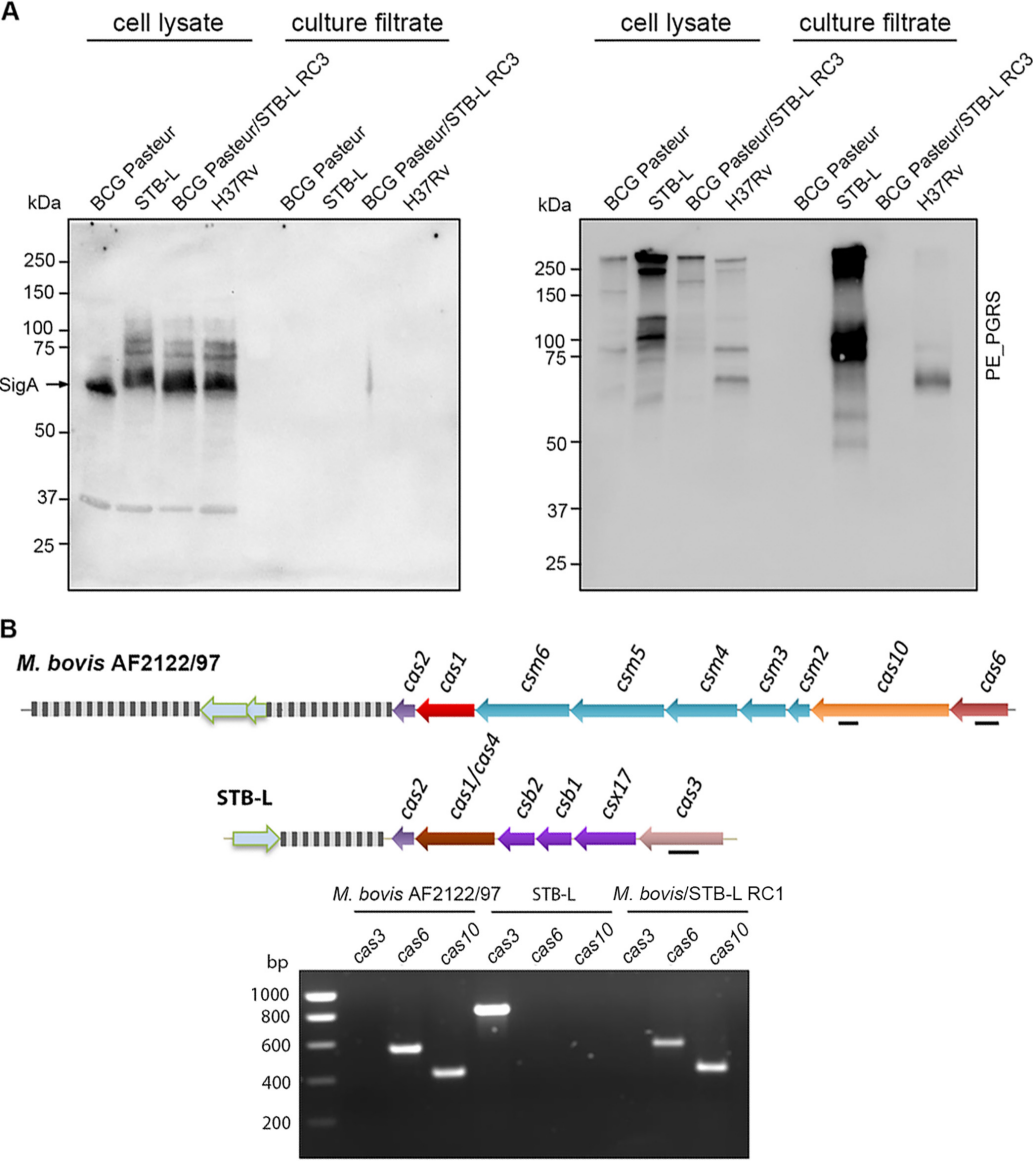
Transfer of large, specific genomic regions and their phenotypic consequences. (A) Analysis of PE_PGRS proteins in whole-cell lysates and culture filtrates of the BCG Pasteur donor, the STB-L recipient, BCG Pasteur/STB-L RC3, and M. tuberculosis H37Rv by immunodetection using anti-PE_PGRS antibodies. Due to the absence of *ppe38-ppe71*, the BCG Pasteur donor strain as well as the recombinant strain BCG Pasteur/STB-L RC3 do not secrete PE_PGRS proteins. SigA was used as a loading and cell integrity control. (B) Schematic representation of the type III-A CRISPR-Cas system found in M. bovis and the type I-C CRISPR-Cas system found in STB-L. PCR analysis of the presence of signature *cas* genes of the respective CRISPR-Cas systems (*cas6* and *cas10* for type III-A and *cas3* for type I-C) in the M. bovis AF2122/97 donor, the STB-L recipient, and M. bovis/STB-L RC1 was performed. Black bars depict the regions amplified by PCR.

Another recombinant clone had received a part of the BCG Pasteur genome that spans the section where the deletion of the region of difference RD5 had occurred in BCG (50, 51) and thus lacked the *plcABC* and *ppe38-ppe71* genes (Fig. 6D; Fig. S2D and Table S1). Previous studies reported that a functional copy of *ppe38*, or its almost identical homologue *ppe71*, was required for the export of proteins with characteristic Pro-Glu (PE) or Pro-Pro-Glu (PPE) motifs, belonging to the PPE major polymorphic tandem repeat (PPE-MPTR) and PE polymorphic GC-rich-repetitive sequence (PE_PGRS) subfamilies, respectively, via the cognate ESX-5 type VII secretion system (51, 52). Accordingly, Western blot analysis of the recombinant clone carrying the BCG Pasteur RD5-deleted region indeed showed a secretion defect for PE_PGRS proteins (Fig. 7A).

Moreover, in several other recombinants, additional donor-specific regions were transferred to the recipient (Fig. S2 and Table S1), with one such example being the transfer of a large segment of the M. bovis AF2122/97 chromosome (coordinates 3060816 to 3103811 of the M. bovis AF2122/97 reference genome) to the STB-L recipient (Fig. 2, bottom left). Strikingly, the transferred fragment spans the entire M. bovis type III-A system CRISPR-Cas locus, which differs strongly from the type I-C CRISPR-Cas locus of STB-L. Given that the CRISPR-Cas loci in both donor and recipient strains occupy the equivalent genomic region (28), the resulting recombinant strain now contains the type III-A CRISPR-Cas system instead of the type I-C CRISPR-Cas system (Fig. 7B). These two examples underline the great potential of the observed DNA transfer to create new genetic combinations and genomic variations, which are important drivers of bacterial evolution.

### What determines mating identity in slow-growing mycobacteria?

In an attempt to identify additional recipient strains, we performed a series of mating assays (Table 1) in which strains STB-G and STB-I reproducibly provided recombinants when used as recipients (Fig. S3 and Table S1), in contrast to M. kansasii, *M. canettii* STB-A, M. tuberculosis H37Rv, and M. bovis AF2122/97, which did not generate Km^r^ Hyg^r^ clones despite identical experimental setups.

**TABLE 1:** Mating pairs with recipient strains other than STB-L^*a*^

Donor	Transfer	Recipient
STB-K	No	M. bovis
STB-D	No	

STB-K	No	M. tuberculosis H37Rv
M. africanum 65	No	
M. bovis	No	

STB-K	No	STB-A
STB-L	No	
M. africanum 65	No	
M. bovis	No	

STB-A	No	STB-E
STB-D	No	
STB-L	No	
STB-G^*b*^	←	

STB-L^*b*^	←	STB-K
STB-G^*b*^	←	
M. bovis	No	

STB-K	→	STB-G
STB-D Δ*eccD_1_*	→	
STB-L	→	
*M. microti*	→	

STB-D	→	STB-I
STB-L	→	

STB-A	No	M. kansasii
STB-D	No	

a A series of mating pair combinations were tested, some of which provided recombinants. The direction of transfer is indicated by the orientation of the arrows, and “No” specifies that no recombinants were obtained despite the same experimental setup. At least two independent experiments were performed for each mating pair.

b When using the STB-L donor strain with the STB-K recipient strain, the resulting recombinants were shown to be STB-L that had received the pMRF1-dsRed plasmid. Kan^r^ Hyg^r^ strains were also obtained from mating the STB-G donor strain with the STB-K and STB-E recipient strains and were shown to be STB-G that had received pMRF1-dsRed.

10.1128/mBio.00965-21.3 FIG S3 Circular representation of selected recombinant genomes when STB-G and STB-I were used as recipient strains. From the outer to the inner circle are (i) the density of detected variants calculated in 5-kb nonoverlapping windows between the recombinant and donor strains (orange), (ii) the donor strain reference genome (red), (iii) the best-scoring hits identified between the recombinant and donor strains (light red), (iv) the assembled recombinant genome (red, region assigned to the donor strain; blue, region assigned to the recipient strain; white, region of unknown origin), (v) the best-scoring hits identified between the recombinant and recipient strains (light blue), (vi) the recipient strain reference genome (blue), and (vii) the density of detected variants calculated in 5-kb nonoverlapping windows between the recombinant and recipient strains (purple). Black bars correspond to gap regions. Coordinates are indicated in megabases. RC, recombinant. Download FIG S3, PDF file, 1.3 MB. Copyright © 2021 Madacki et al. 2021 Madacki et al. https://creativecommons.org/licenses/by/4.0/ This content is distributed under the terms of the Creative Commons Attribution 4.0 International license.

Interestingly, several double-antibiotic-resistant colonies were also obtained in one cross in which STB-L carrying the integrative vector pNIP48 was used as a potential donor strain and STB-K-pMRF1-dsRed was used as a potential recipient; however, WGS of two randomly selected double-resistant clones showed that transfer occurred in the opposite direction of the one expected; i.e., the STB-L::pNIP48 strain had received the pMRF1-dsRed plasmid from strain STB-K, thereby gaining resistance to kanamycin, and in one of the clones, besides the plasmid, additional STB-K-derived chromosomal DNA was transferred (Fig. S4). Similarly, transfer of the pMRF1-dsRed plasmid had occurred when strain STB-G was used as a donor with STB-K or STB-E as a recipient, where in the case of recombinant STB-G/STB-E RC1, segments of chromosomal DNA from the STB-E recipient were also transferred to STB-G (Fig. S4). The transfer of pMRF1-dsRed, an episomal pAL5000-derived plasmid (53), was previously observed in M. smegmatis (26) at a very low frequency, suggesting that the transfer of DNA between mycobacterial strains might not be fully restricted to chromosomal DNA but rather might be dependent on the genotype or phenotype of the recipient strain.

10.1128/mBio.00965-21.4 FIG S4 Circular representation of two recombinants where the STB-L donor strain received the pMRF1-dsRed plasmid from the STB-K recipient strain, one of which also contains STB-K chromosomal DNA segments (top left), as well as recombinants STB-G/STB-K RC1 and STB-G/STB-E RC1, where the STB-G donor had received the pMRF1-dsRed plasmid and, in the case of STB-G/STB-E RC1, chromosomal DNA segments from the STB-E recipient (bottom right). From the outer to the inner circle are (i) the density of detected variants calculated in 5-kb nonoverlapping windows between the recombinant and donor strains (orange), (ii) the donor strain reference genome (red), (iii) the best-scoring hits identified between the recombinant and donor strains (light red), (iv) the assembled recombinant genome (red, region assigned to the donor strain; blue, region assigned to the recipient strain; white, region of unknown origin), (v) the best-scoring hits identified between the recombinant and recipient strains (light blue), (vi) the recipient strain reference genome (blue), and (vii) the density of detected variants calculated in 5-kb nonoverlapping windows between the recombinant and recipient strains (purple). Black bars correspond to gap regions. Coordinates are indicated in megabases. Each recombinant is denoted as the integrative vector-containing donor/DsRed-producing recipient. Download FIG S4, PDF file, 1.0 MB. Copyright © 2021 Madacki et al. 2021 Madacki et al. https://creativecommons.org/licenses/by/4.0/ This content is distributed under the terms of the Creative Commons Attribution 4.0 International license.

Importantly, among donor strains that were able to provide recombinants with STB-G and STB-I recipients was strain STB-L, and an additional cross between the STB-G donor and the STB-L recipient also resulted in recombinants, suggesting that bidirectional transfer is possible between these two strains (Fig. S5).

10.1128/mBio.00965-21.5 FIG S5 Circular representation of recombinants showing bidirectional transfer between the STB-G and STB-L strains. From the outer to the inner circle are (i) the density of detected variants calculated in 5-kb nonoverlapping windows between the recombinant and donor strains (orange), (ii) the donor strain reference genome (red), (iii) the best-scoring hits identified between the recombinant and donor strains (light red), (iv) the assembled recombinant genome (red, region assigned to the donor strain; blue, region assigned to the recipient strain; white, region of unknown origin), (v) the best-scoring hits identified between the recombinant and recipient strains (light blue), (vi) the recipient strain reference genome (blue), and (vii) the density of detected variants calculated in 5-kb nonoverlapping windows between the recombinant and recipient strains (purple). Black bars correspond to gap regions. Coordinates are indicated in megabases. RC, recombinant. Download FIG S5, PDF file, 1.0 MB. Copyright © 2021 Madacki et al. 2021 Madacki et al. https://creativecommons.org/licenses/by/4.0/ This content is distributed under the terms of the Creative Commons Attribution 4.0 International license.

## DISCUSSION

HGT is undoubtedly an important factor in bacterial evolution, and this importance is even more pronounced when antibiotic resistance or virulence genes are being disseminated (1). For the tuberculosis-causing mycobacteria, the question about ongoing HGT remains an open subject, with members of the MTBC showing an overall clonal population structure (27, 54) and members of the closely related *M. canettii* clade depicting a recombinogenic population structure (28). In previous work, *M. canettii* strain STB-L was identified as a recipient strain of chromosomal DNA fragments transferred from a single donor strain named STB-A (26). Here, we experimentally demonstrate the transfer of chromosomal DNA from a wide range of MTBC member strains to STB-L and also identify *M. canettii* strains STB-G and STB-I as recipients that are capable of integrating donor DNA segments into their genome. However, despite numerous attempts, no other strains were found capable of acting as recipient strains in our mating assay, suggesting that strains STB-G, STB-I, and STB-L are characterized by particular features that allow them to take up and recombine donor DNA segments. This finding opens new perspectives for future studies to identify potential common genetic factors that characterize recipient phenotypes. The results also show that M. tuberculosis and M. bovis strains, representing the globally most widely distributed pathogens among the MTBC members, apparently lack the ability to act as recipients, whereas they can still act as donors. This scenario is in agreement with the stable clonal phylogeographic lineages that are formed within the MTBC, in which the vertical transfer of genes, mutations, and deletions defines the genotypes of the daughter generations and where loss-of-function mutations cannot be repaired by HGT (25, 27, 54, 55).

A closer look shows that only three (*msmeg_0069* [*espJ*], *msmeg_0071* [*espK*], and *msmeg_0076* [*espB*]) out of six previously reported so-called *mid* (mating identity) genes (9) have putative orthologues in tuberculosis-causing mycobacteria (26) and that this region does not differ in gene content between donor and recipient tubercle bacilli. Moreover, we have seen that strains STB-G and STB-L are capable of bidirectional transfer, similar to M. smegmatis isolates PM5/mc^2^874 and Jucho (4, 6), a phenomenon that deserves attention, as mating-type switching has been well described only in Saccharomyces cerevisiae (56).

Conjugation in diderm bacteria is usually mediated by type IV secretion systems or similar large transport machineries located in the cell envelope of donor cells (57–59). Interestingly, screening of donor mutants deficient in conjugal transfer in M. smegmatis did not identify any transport proteins essential for this process, and rather paradoxically, a deletion in the type VII secretion system ESX-1 of the M. smegmatis donor was found to enhance transfer (18). In contrast to these previous reports, here, we show that when the ESX-1 secretion machinery is disabled in *M. canettii* or M. tuberculosis donors, there is no hyperconjugative phenotype; moreover, in all mutants tested, there is a tendency toward a lower transfer efficiency. Owing to the relatively high variability of individual points in these assays, it is difficult to conclude whether the observed changes are significant, although the pattern is clearly present in all studied mutant strains. The use of a recombinant BCG Pasteur 2F9 strain carrying an *esx-1* locus from M. tuberculosis (24), however, did not show any significant differences in transfer efficacy compared to the BCG Pasteur parental strain (Fig. 5A). As the complementation of BCG with cosmid pRD1-2F9 encoding ESX-1 from M. tuberculosis was usually associated with a strong change of phenotype (24), the similarities in transfer efficacies of recombinant and parental BCG strains observed here further suggest that ESX-1 is not involved in the transfer process in tubercle bacilli. In previous work, the integration of pRD1-2F9 into different M. smegmatis hyperconjugative ESX-1 knockout mutants showed variable levels of complementation, a result which was interpreted by those authors as the functional equivalence of the ESX-1 regions from M. smegmatis and M. tuberculosis (18). Taken together with our results, it is now apparent that despite the similarities in the secreted WXG proteins EsxA and EsxB (60, 61), a clear difference between ESX-1 involvement in HGT in M. smegmatis and tuberculosis-causing mycobacteria exists. In M. smegmatis, recipient ESX-1 and ESX-4 secretion systems were reported to be indispensable for conjugation (19–21), a finding that is in stark contrast to our results for tubercle bacilli, where we did not observe an involvement of a functional ESX-1 system whatsoever, neither for the donor nor for the recipient strains.

In our experiments, we also included two NTM strains belonging to species that are relatively closely related to the MTBC: M. kansasii and M. lacus (44). However, unlike all tested MTBC and *M. canettii* strains, these strains did not provide any recombinants when used as putative donors together with the well-established recipient strain STB-L. Similarly, when M. kansasii was used as a recipient strain with two potential *M. canettii* donor strains, no recombinants were obtained. Given these results, it is tempting to speculate that, although probably being a process happening in most mycobacterial species, HGT in tubercle bacilli depends on factors involved in interstrain transfer as well as the degree of sequence similarity of fragments that may recombine with the recipient’s genome. Our results thus underline the classification of *M. canettii* and the MTBC members into the group of tuberculosis-causing mycobacteria/tubercle bacilli, within which all members could act as donors, in contrast to the NTM species *M. lacus* and M. kansasii. The roles of additional factors deemed essential for transfer in M. smegmatis, such as the above-mentioned ESX-4 system or the Lsr2 and LpqM proteins (62, 63), in HGT in tubercle bacilli remain to be examined. Such studies will also determine whether the ESX-4 system in tubercle bacilli is fully functional, as it lacks the EccE component, which for other ESX systems is an essential part of the secretion apparatus (12–14). There are only a few mycobacterial species known to harbor an ESX-4 system comprising the EccE component, like, for example, M. abscessus (64), for which an important role of ESX-4 in *in vivo* growth was described (65).

After transfer, chromosomal DNA originating from the donor is exchanged with the corresponding sequences in the recipient cell by homologous recombination, and this process seems to be dependent on the presence of recipient *recA* and *recB* in M. smegmatis (8). The same study showed that donor *recA* mutants have a higher transfer efficiency, and it was hypothesized that in this case, eventual breaks in the DNA would not be repaired efficiently; therefore, more fragments would be available for transfer. In the context of our results, M. bovis AF2122/97 and BCG have a truncated *recB* gene, which encodes a subunit of the helicase-nuclease RecBCD (46), and in addition, BCG Russia is known to be a *recA* mutant (66). While M. bovis AF2122/97 generated the highest transfer efficiency of all donors used in our study, the *recA* mutation in BCG Russia did not seem to positively affect the transfer efficiency compared to BCG Pasteur (Fig. 5A). This example again shows that observations made for the fast-growing mycobacterial model organism M. smegmatis do not necessarily also apply to the MTBC. In addition, mycobacteria also contain additional repair systems for repairing double-strand breaks, such as nonhomologous end joining (NHEJ) catalyzed by Ku and DNA ligase D (LigD), the AdnAB helicase-nuclease, and single-strand annealing (SSA) (67, 68). It remains to be determined if any of these other repair mechanisms are involved in the exchange of the donor-transferred DNA fragments into the genomes of tubercle bacilli.

Additional important questions arise as we show here that various MTBC members can act as DNA donors. The observation that the region of difference RD5, lacking *plcABC* and *ppe38*-*ppe71* genes, which is characteristic of the M. bovis/M. bovis BCG (50) lineage and certain *M. microti* (25) and M. africanum (32) strains, was transferred from BCG Pasteur to STB-L is a particularly striking example that such transfer may also have phenotypic consequences. Indeed, the absence of *ppe38*-*ppe71* genes has been described as being responsible for the loss of secretion of a large class of mycobacterial surface proteins of the PE_PGRS and PPE-MPTR subgroups (52) (Fig. 7A) that also potentially impact the virulence of the resulting strain (51, 52). Similarly, the transfer of a completely different CRISPR-Cas operon from M. bovis to *M. canettii* (Fig. 7B) underlines the genome dynamics that prevail inside the population of tubercle bacilli and in particular within the strains that can still act as recipients. Indeed, during previous genome analyses of various *M. canettii* strains, four different CRISPR-Cas systems of type III-A, type I-C, type I-C-var, and type I-E were identified (28, 69), whereas members of the MTBC are characterized by a type III-A locus.

The examples of the transfer of the RD5 and CRISPR-Cas regions also nicely demonstrate that any chromosomal region may be transferred, even if a potential loss or gain of function is connected with it. It is tempting to speculate that in such a scenario, the transfer of drug resistance mutations might also be possible. While we do not dispose of an example of such transfer, as *M. canettii* recipient strains with the exception of clade-specific resistance to pyrazinamide (70) usually show an overall drug-sensitive phenotype, it should be mentioned that coinfection with M. tuberculosis and *M. canettii* was described in two patients in Djibouti (71), thereby opening the theoretical possibility of such transfer. Moreover, a recent study identified two early-branching and rare MTBC strains, defined as lineage 8 strains (36), both of which showed uncommon multidrug-resistant (MDR) genotypes. It would indeed be interesting to examine such strains that are situated phylogenetically at the basis of the MTBC to determine if they had *M. canettii*-like abilities to act as recipient strains or if their surprising MDR genotypes were simply caused by common prolonged exposure to antibiotic treatment.

In conclusion, in the current study, we have identified important new features of HGT between strains of tuberculosis-causing mycobacteria. First, we found that all tested MTBC and *M. canettii* strains were capable of acting as DNA donors transferring random chromosomal DNA fragments to recipient strains STB-L, STB-G, and/or STB-I, thereby showing that HGT between tubercle bacilli is still active although restricted and directed toward selected *M. canettii* strains as recipients. Second, we have collected converging evidence from different experiments showing that the ESX-1 system is not involved in the observed DNA transfer events among tubercle bacilli. This finding clearly distinguishes HGT between tubercle bacilli from ESX-1-dependent transfer mechanisms, previously reported for M. smegmatis strains, and thereby opens new, exciting questions about the potential mechanisms involved in HGT in mycobacterial evolution. Our results are also in agreement with recent reports on the recombinogenic population structure observed in mycobacterial species that are naturally devoid of ESX-1 secretions systems, such as the slow-growing species Mycobacterium avium (72) or the fast-growing species Mycobacterium abscessus (73). This situation argues that ESX-1-independent HGT is widely distributed among mycobacteria and likely plays a key role in shaping mycobacterial evolution. Besides the canonical gene transfer mechanisms of transformation, transduction, and conjugation, a fourth way of HGT was recently suggested to be named vesiduction (74). The latter form of DNA transfer via extracellular vesicles (EVs) is still underappreciated, and it will certainly be worth exploring in future research whether some of the transfer events described in this work might rely on such a type of transfer, especially as EVs have been described in mycobacterial species, including Mycobacterium ulcerans (75), M. avium (76), and M. tuberculosis (77, 78). Our findings thereby constitute the scientific basis for novel searching strategies. Our results also emphasize that slow-growing mycobacterial pathogens may differ quite strongly from the more distantly related, nonpathogenic model organisms that are often used in mycobacterial research. This feature also seems to apply to the mechanisms that are driving their evolution, justifying the need to work with the actual pathogens in order to draw conclusions in their regard.

## MATERIALS AND METHODS

### Bacterial strains and media.

Mycobacterial strains (see Table S2 in the supplemental material) were routinely grown on Middlebrook 7H11 agar medium (Difco) supplemented with 10% oleic acid-dextrose-catalase (OADC; Difco) and 0.5% glycerol or in Middlebrook 7H9 liquid medium (Difco) supplemented with 10% acid-dextrose-catalase (ADC; Difco), 0.2% glycerol, and 0.05% Tween 80, with the addition of 0.2% pyruvate for M. africanum, *M. microti*, M. orygis, M. pinnipedii, M. caprae, and M. bovis. Escherichia coli strain DH10B was used for cloning purposes and was grown in Luria-Bertani (LB) liquid medium or LB agar plates at 37°C. When required, the following antibiotics were added: kanamycin (25 μg/ml for mycobacteria other than M. kansasii and 50 μg/ml for E. coli and M. kansasii), hygromycin (50 μg/ml for mycobacteria and 200 μg/ml for E. coli), zeocin (25 μg/ml), and gentamicin (25 μg/ml).

10.1128/mBio.00965-21.7 TABLE S2 Mycobacterial strains used in this work. Download Table S2, PDF file, 0.2 MB. Copyright © 2021 Madacki et al. 2021 Madacki et al. https://creativecommons.org/licenses/by/4.0/ This content is distributed under the terms of the Creative Commons Attribution 4.0 International license.

### Mating assays.

In order to introduce antibiotic markers, donor strains were transformed with an integrative vector, pYUB412 (41) or pNIP48 (79), carrying a hygromycin cassette, and recipient strains were transformed with an episomal plasmid, pMRF1-dsRed, carrying a kanamycin cassette and enabling the production of the DsRed fluorophore, making these colonies distinguishable from donor colonies by the naked eye. Mating assays were performed as described previously (6, 26). Briefly, donor and recipient cultures were grown at 37°C to the late exponential phase when cells were harvested and resuspended in fresh medium without antibiotic to an optical density at 600 nm (OD_600_) of 1.5. Five hundred microliters of donor and 500 μl of recipient cell suspensions were mixed, the mixture was passed through a 0.45-μm filter (Merck Millipore), and the filter was placed with the bacterial side up on a 7H11 agar plate without antibiotics, which was incubated for 7 days at 37°C. After incubation, bacteria were scraped off the filter and thoroughly resuspended in fresh liquid medium, and recombinants were selected on 7H11 plates containing kanamycin and hygromycin. For assays where the conjugation efficiency was to be determined, these bacterial suspensions were also plated on single-antibiotic-containing plates, and the efficiency was calculated as the number of recombinants per recovered donor or recipient cell. In order to exclude spontaneous resistance to hygromycin, recipient monocultures were also filtered and plated on double-antibiotic plates (Fig. 1A). The presence of kanamycin and hygromycin cassettes in recombinant clones was verified in colony lysates by PCR using primers listed in Table S3.

10.1128/mBio.00965-21.8 TABLE S3 Primers used in this work. Download Table S3, PDF file, 0.2 MB. Copyright © 2021 Madacki et al. 2021 Madacki et al. https://creativecommons.org/licenses/by/4.0/ This content is distributed under the terms of the Creative Commons Attribution 4.0 International license.

### Construction of ESX-1 deletion mutants.

For disrupting *eccD_1_* in STB-A, STB-D, and STB-K, the recombineering system (80) was used, while the ts-*sacB* method that is based on a temperature-sensitive mycobacterial origin of replication (ts-*oriM*) and *sacB* as a counter-selective marker (81) was used for disrupting this gene in STB-L. First, an allelic exchange substrate (AES) was prepared for each strain using a three-step PCR procedure (82), which consisted of amplifying a Zeo^r^ cassette as well as 500-bp regions upstream and downstream of the *eccD_1_* ORF using primers with overlapping sequences (Table S3) and then joining the three resulting fragments in a single PCR with equimolar amounts of each, using the forward primer of the upstream fragment and the reverse primer of the downstream fragment. PCR products of the expected size (1,638 bp) were isolated from an agarose gel and ligated into the pJET1.2 vector (Thermo Fisher) used for subcloning, and the correct sequence was confirmed by Sanger sequencing.

In the case of STB-A, STB-D, and STB-K, each strain was first transformed with the plasmid pJV53 (kindly provided by Graham F. Hatfull, University of Pittsburgh, Pittsburgh, PA, USA), and transformants were selected on kanamycin-containing plates at 37°C. The resulting strains were grown in 7H9 liquid medium supplemented with 0.2% succinate and 0.05% Tween 80 and induced at an OD_600_ of 0.25 with 0.2% acetamide for 24 h at 37°C. Cells were electroporated with 500 to 700 ng of the linear double-stranded DNA (dsDNA) AES, and recombinants were selected on plates containing kanamycin and zeocin at 37°C. Individual transformants were verified for *eccD_1_* deletion by PCR, and once the correct deletion was confirmed, the pJV53 plasmid was cured by passaging on zeocin plates.

For the ts-*sacB* method, the linear AES was amplified using primers introducing end restriction sites for SpeI/XbaI (Table S3), which were used for cloning into the thermosensitive vector pPR27 digested with the same restriction endonucleases. The resulting construct was subsequently electroporated into STB-L, and zeocin-resistant transformants were selected on 7H11 agar at 32°C and further propagated in 7H9 liquid medium containing zeocin at 32°C. Saturated cultures were then plated onto 7H11 zeocin plates supplemented with 10% OADC, 0.5% glycerol, and 5% sucrose, which were grown at 39°C, and colonies were screened by PCR for successful double crossover.

### Whole-genome sequencing and assembly.

Genomic DNA was extracted from selected putative recombinants, as well as from each donor and recipient strain, as previously described (83), and the resulting DNA samples were used for library preparation using the TruSeq DNA PCR-free library prep kit (Illumina) or the Nextflex PCR-free DNA-Seq kit for Illumina (Bioo Scientific). DNA sequencing was performed at the Biomics platform of the Institut Pasteur (Paris, France) using a paired-end 100-bp run on a HiSeq 2500 device (Illumina) or a paired-end 150-bp run on a NextSeq 500 device (Illumina). Sequencing reads were first trimmed using Trimmomatic v0.36 (84) (parameters LEADING 28, TRAILING 28, SLIDINGWINDOW windowSize 5, requiredQuality 28, MINLEN 50, and AVGQUAL 28). Complete read pairs were then *de novo* assembled using SPAdes v3.10.1 (85) (parameters --careful, -k 21,33,55 for HiSeq reads or -k 21,33,55,77 for NextSeq reads, and --phred-offset 33). The contigs thus generated were finally organized using MeDuSa v1.6 (86) (default parameters) and compared to the corresponding reference genome of each donor or recipient strain (see Table S4 in the supplemental material for details) (25, 28, 32, 45, 47, 87–89).

10.1128/mBio.00965-21.9 TABLE S4 List of accession numbers for the genome data used or generated in this study. Download Table S4, PDF file, 0.2 MB. Copyright © 2021 Madacki et al. 2021 Madacki et al. https://creativecommons.org/licenses/by/4.0/ This content is distributed under the terms of the Creative Commons Attribution 4.0 International license.

### Whole-genome comparison of recombinants.

To distinguish donor-derived DNA from recipient DNA within each sequenced recombinant, the following two approaches were used based on the whole-genome alignment of the reconstructed recombinant sequence against donor and recipient strain sequences and the detection of mismatches.

(i) Each assembled recombinant genome was aligned against the reference genome of the corresponding donor and recipient strains (Table S4) using NUCmer from the MUMmer package v3.1 (90) (default parameters). Detection of variants (single nucleotide polymorphisms [SNPs] and indels) from the NUCmer alignment was then performed using successively the commands delta-filter (parameters -q and -r), show-coords (parameters -c, -l, and -r), and show-snps (parameters -C, -l, and -r). Ambiguous variants that were located within repetitive sequences, namely, genes encoding proteins of the Pro-Glu (PE), Pro-Pro-Glu (PPE), and PE_PGRS (polymorphic GC-rich sequence) families, mobile elements, and repeat regions, were removed. Nonspecific variants that were detected when comparing the reconstructed genome of control donor and recipient strains against their respective reference sequences (Table S4) were also filtered out. The resulting list of kept variants was then converted into a format readable by ACT (48) for visualization. In addition, the density of filtered variants was calculated in 5-kb nonoverlapping windows.

(ii) Each assembled recombinant genome was cut in 100-bp nonoverlapping windows and compared against the reference genome of the respective donor and recipient strains (Table S4) using BLASTN from BLAST^+^ v2.5.0^+^ (91) (parameters -perc_identity 95, -strand plus, -dust no, and -soft_masking no). Each window was assigned to the corresponding donor or recipient strain according to the best-hit score. In the case of identical bit scores between both donor and recipient strains, the window was assigned to the recipient strain. Successive windows assigned to the same strain were then concatenated to obtain the longest possible continuous sequence. The resulting donor- and recipient-attributed recombinant sequences were finally converted into a format readable by ACT (48) for visualization.

Circular representations of recombinants, variant densities, and donor- and recipient-assigned regions were performed using Circos v0.69-6 (92).

### Western blotting.

Samples were prepared for Western blotting as described previously (45). Briefly, cultures were grown to mid-exponential phase in 7H9 liquid medium supplemented with 10% ADC, 0.2% glycerol, and 0.05% Tween 80, at which point cells were washed with the same medium without ADC but with the addition of 0.2% dextrose. Cultures were left to incubate in this medium for 48 h, cells were harvested, and the supernatants were filtered through a 0.22-μm filter. Cell pellets were washed and resuspended in 1× phosphate-buffered saline (PBS) and lysed using Bead Mill 24 (Fisher Scientific) twice for 45 s at 6 m/s with a 30-s cooling interval, and lysates were filtered using a 0.22-μm filter. Forty micrograms of proteins from both the whole-cell lysate and supernatant fractions was separated by SDS-PAGE on NuPage 10% Bis-Tris gels (Invitrogen) and transferred onto a nitrocellulose membrane using the iBlot dry blotting system (Invitrogen). For immunodetection, anti-PGRS antibody 7C4.1F7 (1:2,000) (the antibody-producing clone was a kind gift from M. J. Brennan, Aeras, Rockville, MD, USA, and purified antibody was a kind gift from W. Bitter, Amsterdam UMC, Amsterdam, The Netherlands) (93) or anti-SigA (1:5,000) (a kind gift from I. Rosenkrands, Statens Serum Institut, Copenhagen, Denmark) was used, followed by horseradish peroxidase (HRP)-conjugated IgG anti-mouse or anti-rabbit secondary antibody (Amersham) (both diluted 1:5,000), respectively.

### Data availability.

Illumina sequencing reads have been deposited in the European Nucleotide Archive (ENA) database under the study accession number PRJEB42505. Individual-run accession numbers are listed in Table S4 in the supplemental material.
