# Supplementary figures and images for: ESX-1-Independent Horizontal Gene Transfer by Mycobacterium tuberculosis Complex Strains
Source: mBio. 2021 May 18;12(3):e00965-21. doi: 10.1128/mBio.00965-21 (PMC8262963; doi:10.1128/mBio.00965-21)

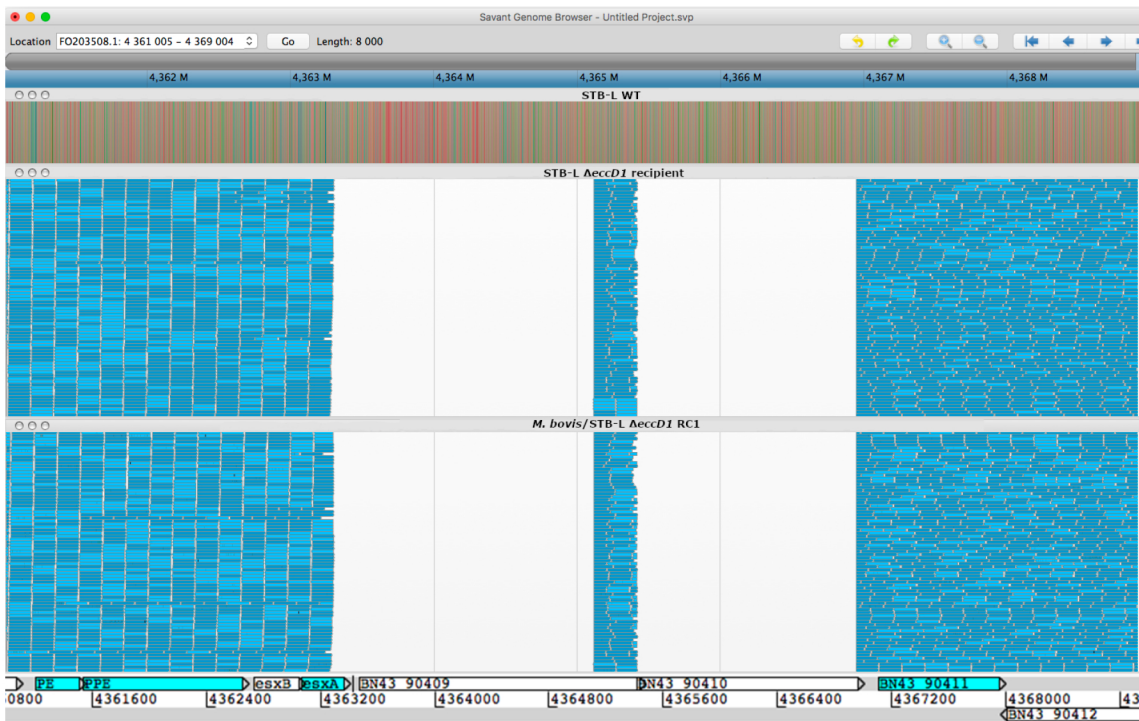

Fig. S1

Supplement: FIG S1 [file mbio.00965-21-sf001.pdf]

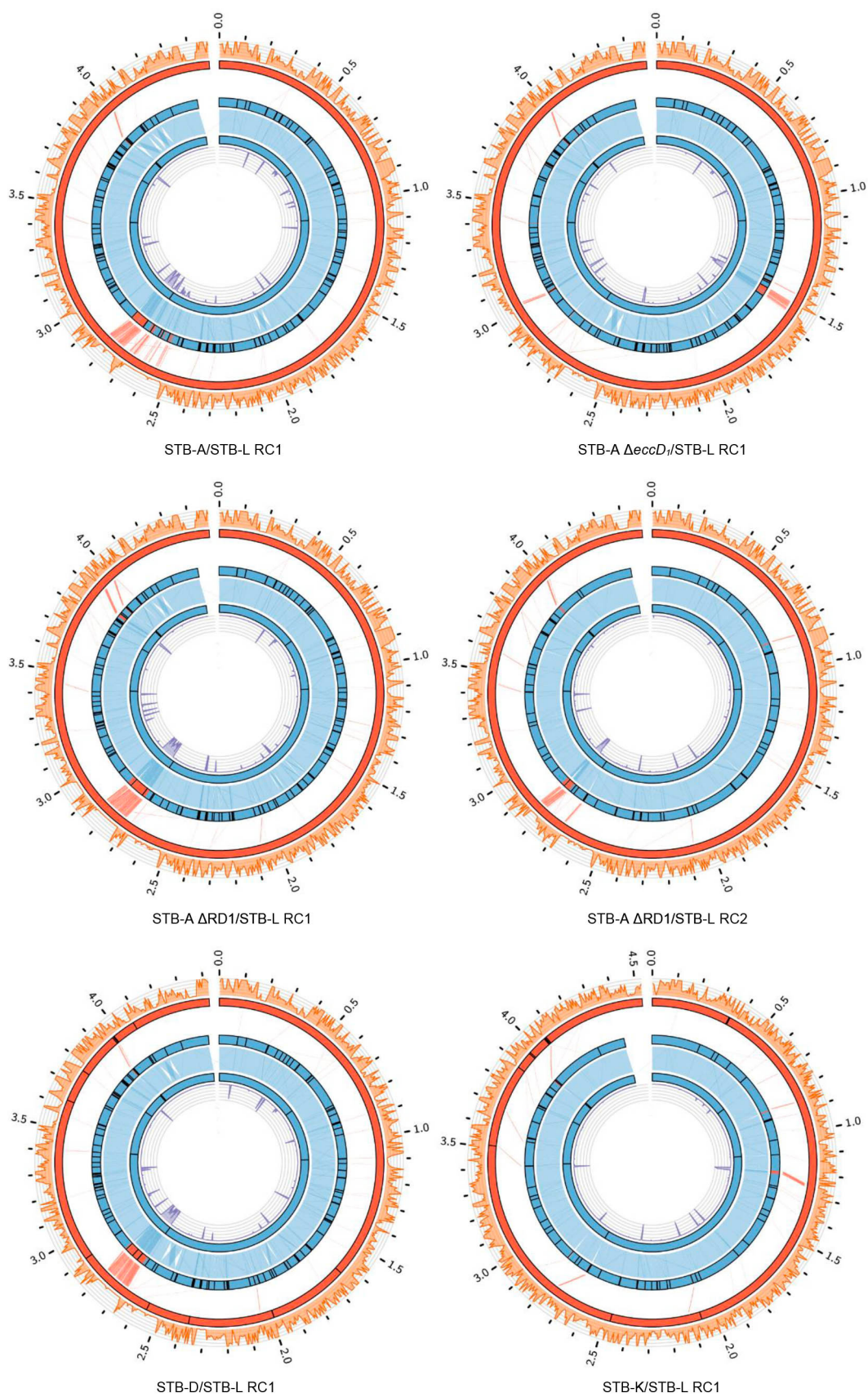

**Fig. S2A**

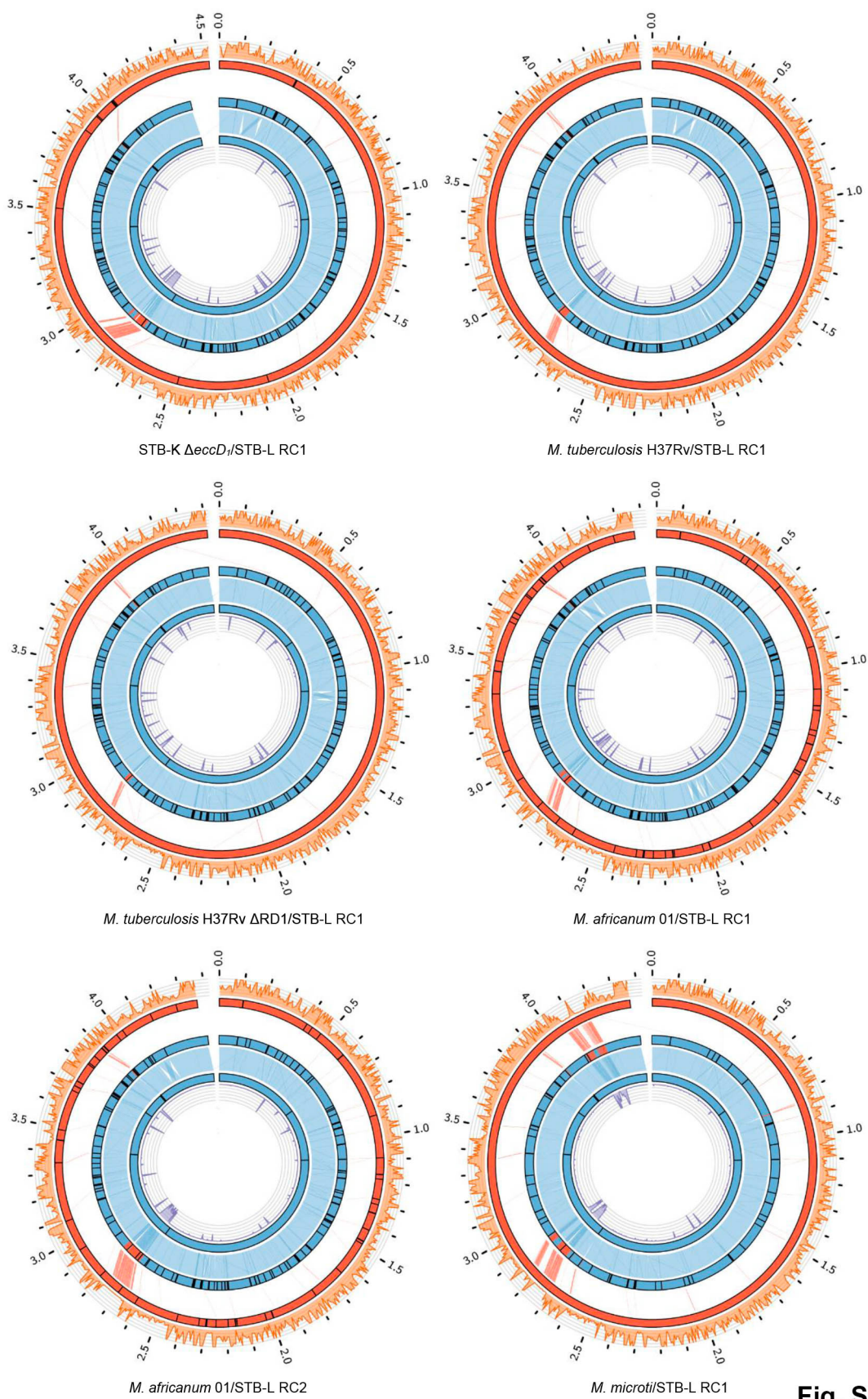

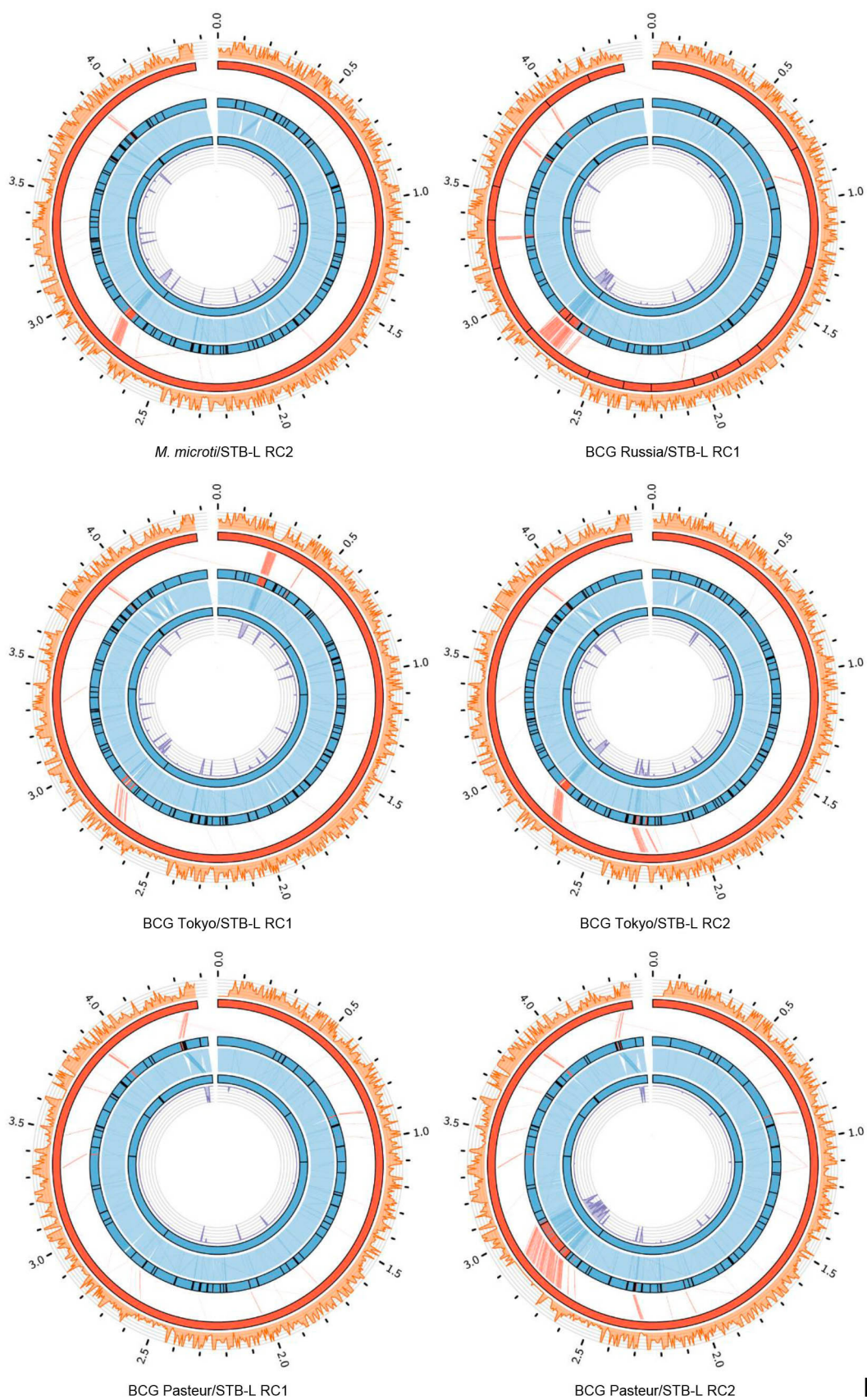

**Fig. S2C**

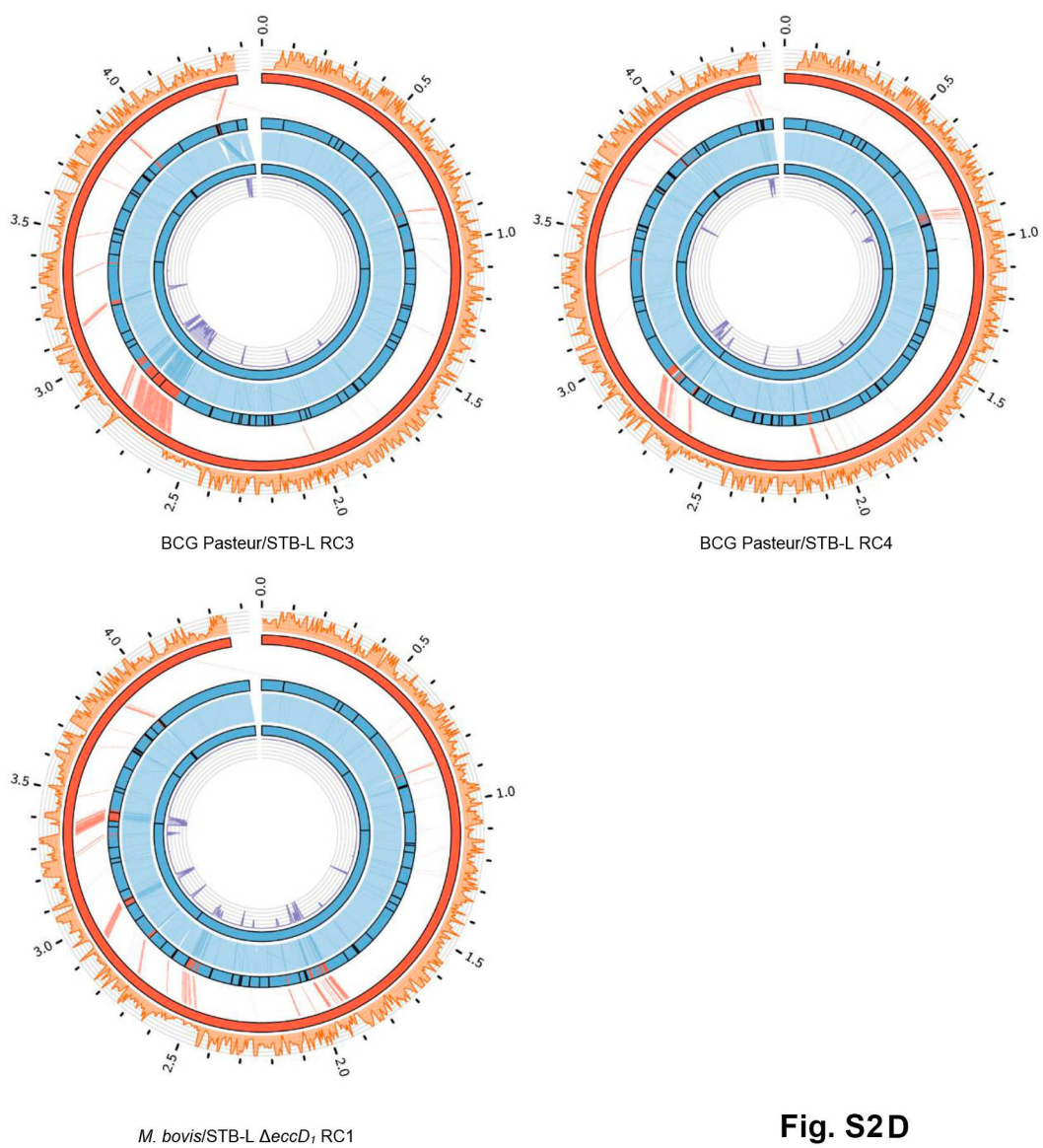

**Fig. S2D**

Supplement: FIG S2 [file mbio.00965-21-sf002.pdf]

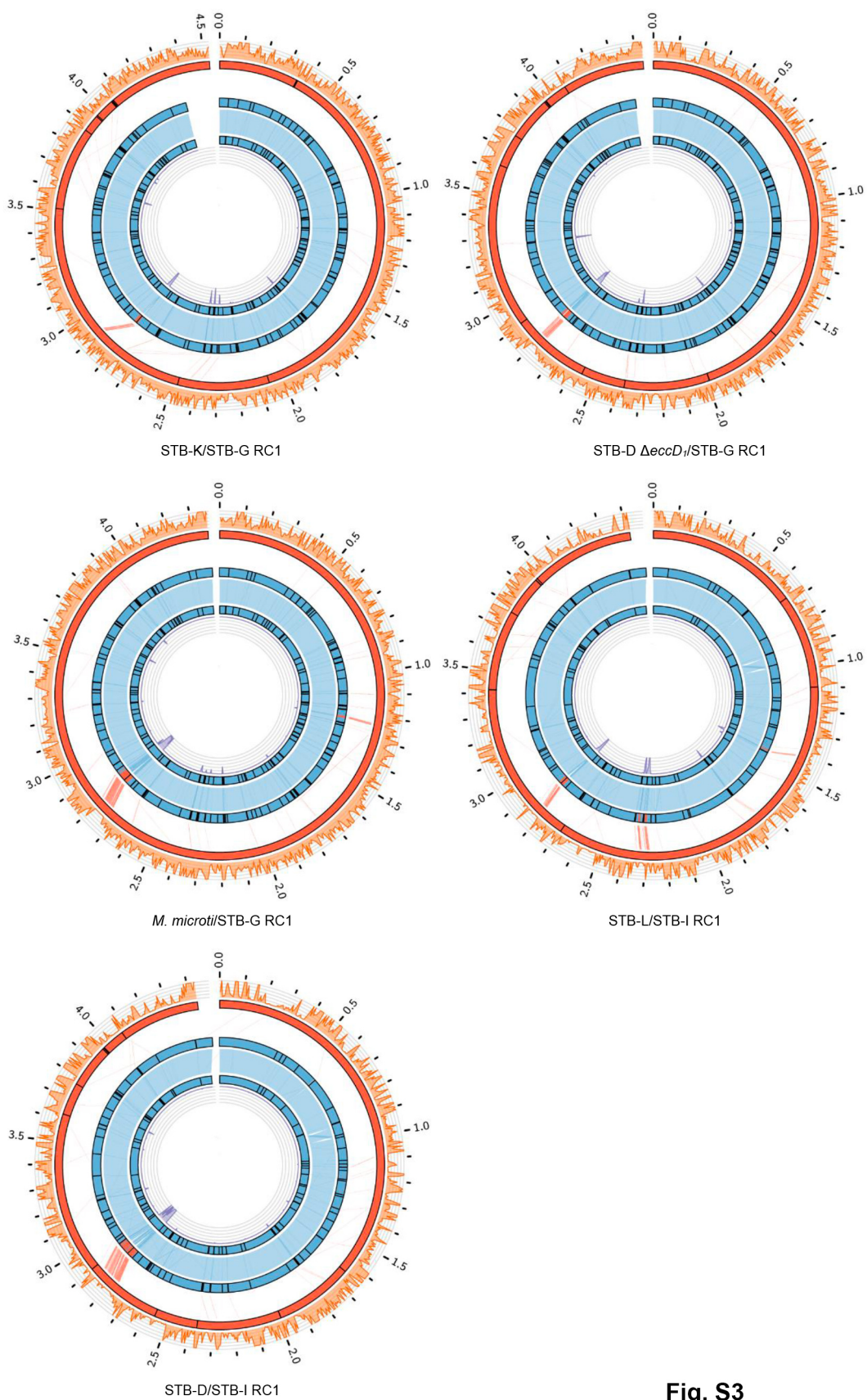

**Fig. S3**

Supplement: FIG S3 [file mbio.00965-21-sf003.pdf]

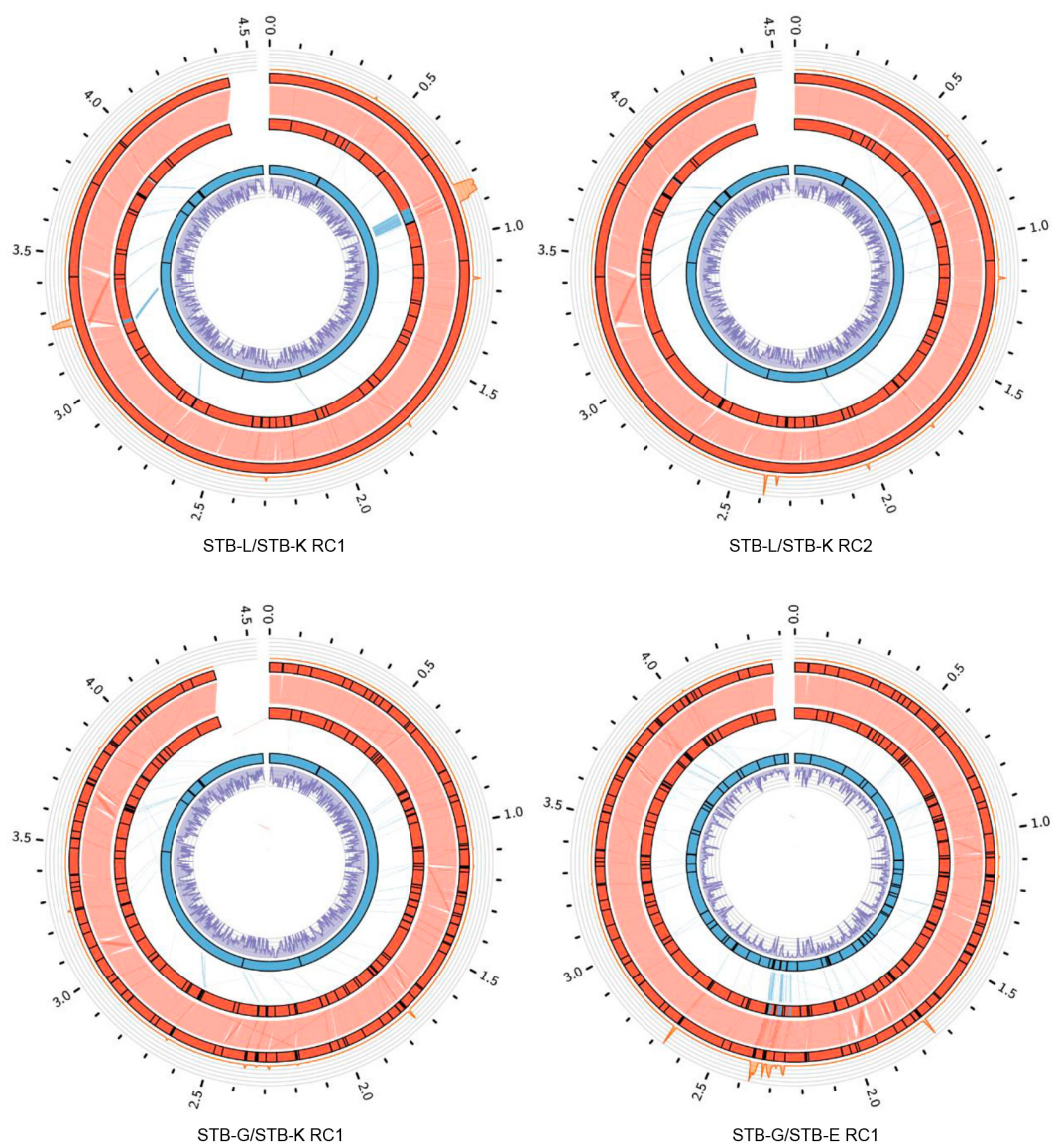

**Fig. S4**

Supplement: FIG S4 [file mbio.00965-21-sf004.pdf]

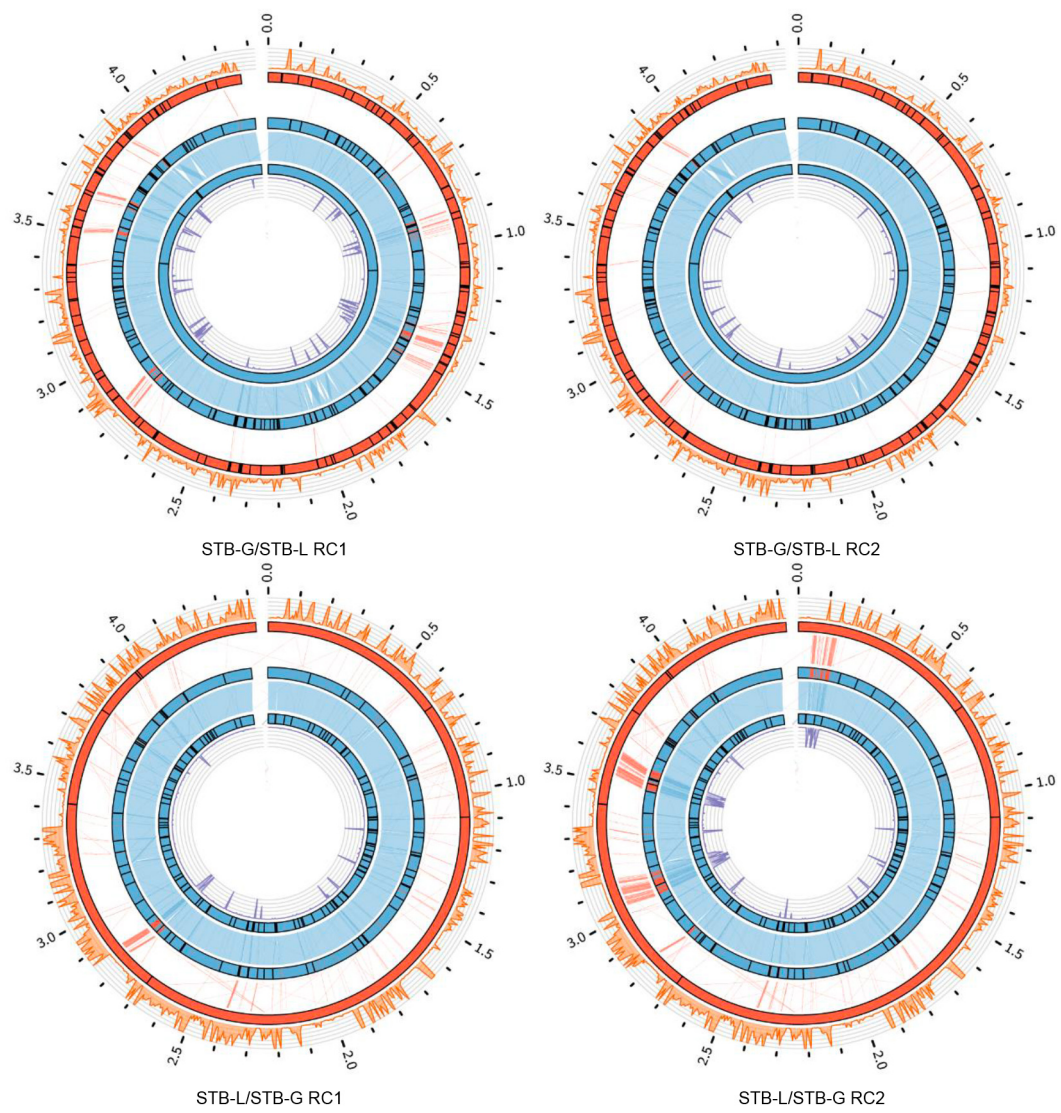

**Fig. S5**

Supplement: FIG S5 [file mbio.00965-21-sf005.pdf]
