# Supplementary material for: ESX-1-Independent Horizontal Gene Transfer by Mycobacterium tuberculosis Complex Strains
Source: mBio. 2021 May 18;12(3):e00965-21. doi: 10.1128/mBio.00965-21 (PMC8262963; doi:10.1128/mBio.00965-21)
Supplement: TABLE S1 [file mbio.00965-21-st001.pdf]

Table S1: List of recombinant strains that were subjected to WGS. Donor-derived sequences were identified based on polymorphism signals between the recombinant sequence and the recipient reference sequence. For each fragment, start and end positions on the recipient reference genome are given. M – regions of microcomplexity; DS – regions where additional donor-specific sequences are present inside the transferred fragment. For each of these fragments, start and end positions on the donor reference genome are also given.

name of Recombinant	number of donor-derived continuous regions	total size in bp	start (recipient reference)	end (recipient reference)	size in bp	details	start (donor reference)	end (donor reference)	size in bp
STB-A/STB-L RC1	8	131253	2561286	2573494	12209				
			2603687	2608223	4537				
			2623276	2632858	9583				
			2675530	2693809	18280				
			2699772	2705834	6063				
			2737593	2746337	8745				
			2747695	2785979	38285				
			2789914	2823464	33551				
STB-A ΔRD1/STB-L RC1	2	133663	2723573	2844175	120603				
			3890590	3903649	13060				
STB-A ΔRD1/STB-L RC2	1	43460	2792761	2836220	43460				
STB-A Δ*eccD1* /STB-L RC1	4	62926	1450948	1454863	3916				
			1496815	1542314	45500				
			2821023	2821986	964				
			2992343	3004888	12546				
STB-D/STB-L RC1	1	122242	2704714	2826955	122242				
STB-K/STB-L RC1	2	17815	1190285	1207819	17535				
			2820813	2821092	280				
STB-K Δ*eccD1* /STB-L RC1	2	82269	2761009	2769779	8771				
			2769780	2770123	344	DS	2836061	2838260	2200
			2770124	2832854	62731				
			2861386	2871808	10423				
M. tuberculosis H37Rv/STB-L RC1	1	45033	2783419	2828451	45033				
M. tuberculosis H37Rv ΔRD1/STB-L RC1	1	21726	2815761	2837486	21726				
M. africanum 65/STB-L RC1	4	193433	2808868	2815033	6166				
			2815240	2816392	1153	M			
			2816383	2905609	89227				
			2916130	3013016	96887				
M. africanum 65/STB-L RC2	4	89914	2763490	2767828	4339	M			
			2768677	2814466	45790				
			2814471	2815956	1486	M			
			2815943	2854241	38299				

M. africanum 01/STB-L RC1	2	165333	2802713	2834145	31433				
			2851238	2876324	25087				
M. africanum 01/STB-L RC2	2	108813	2730856	2739395	8540				
			2745730	2846002	100273				
M. microti /STB-L RC1	4	198703	2779064	2846949	67886				
			2882293	2915185	32893				
			4096164	4145546	49383				
			4168447	4216987	48541				
M. microti /STB-L RC2	1	53505	2776815	2830319	53505				
M. bovis /STB-L RC1	6	389117	2813653	2993564	179912				
			2993710	2994907	1198	M			
			2995345	3013048	17704				
			3038453	3143146	104694				
			3143147	3181225	38079	DS	3060816	3103811	42996
			3181226	3228755	47530				
M. bovis /STB-L RC2	3	124702	2756025	2854267	98243				
			2954646	2957535	2890				
			2962283	2985851	23569				
M. bovis BCG Russia/STB-L RC1	6	159788	2650014	2666805	16792				
			2700162	2712408	12247				
			2715131	2753284	38154				
			2760768	2825837	65070				
			3288533	3305244	16712				
			3750747	3761559	10813				
M. bovis BCG Tokyo/STB-L RC1	5	92231	232722	278520	45799				
			409039	412988	3950				
			2781643	2789899	8257				
			2802304	2827310	25007				
			2839881	2849098	9218				
M. bovis BCG Tokyo/STB-L RC2	3	100506	2270593	2278399	7807				
			2316872	2353219	36348				
			2773391	2829741	56351				
M. bovis BCG Pasteur/STB-L RC1	1	7743	-	-	7743	pYUB412 sequence			
M. bovis BCG Pasteur/STB-L RC2	7	242848	2156594	2158070	1477				
			2331520	2350507	18988				
			2730094	2733819	3726				
			2771254	2799355	28102				
			2811929	2817770	5842	M			
			2817878	2882234	64357				
			2883484	3003839	120356				
M. bovis BCG Pasteur/STB-L RC3	5	236906	2674850	2686043	11194	RD5 deletion			
			2692964	2703164	10201	RD5 deletion			

			2705552	2870994	165443	
			2899243	2933973	34731	
			3222157	3237493	15337	
M. bovis BCG Pasteur/STB-L RC4	6	104509	842171	870378	28208	
			2107055	2121084	14030	
			2693099	2703367	10269	
			2788486	2807091	18606	
			2817222	2817492	271	M
			2820726	2853850	33125	
M. bovis /STB-L *ΔeccD1* RC1	14	179086	1911871	1929351	17481	
			1938031	1945087	7057	
			1955709	1957059	1351	
			1964886	1966857	1972	
			1989349	2000499	11151	
			2107509	2112216	4708	
			2557547	2565900	8354	
			2579530	2619315	39786	
			2820757	2830691	9935	
			3002740	3034107	31368	
			3339979	3341248	1270	
			3351520	3353779	2260	
			3419294	3455143	35850	
			3461371	3467913	6543	
STB-K/STB-G RC1	2	15200	2792788	2802379	9592	
			2804476	2810083	5608	
STB-D Δ*eccD1* /STB-G RC1	2	32226	2773124	2798210	25087	
			2800973	2808111	7139	
M. microti /STB-G RC1	2	59777	2778084	2833935	55852	
			2845114	2849038	3925	
STB-G/STB-L RC1	14	196470	619179	621987	2809	
			869341	874984	5644	
			904946	943663	38718	
			1349078	1367150	18073	
			1387896	1408619	20724	
			1415111	1429902	14792	
			1480134	1505871	25738	
			2023398	2024604	1207	
			2812814	2838211	25398	
			2851189	2862017	10829	
			3543335	3560055	16721	
			3676159	3678508	2350	
			3698645	3710229	11585	
			4348296	4350177	1882	

STB-G/STB-L RC2	1	24333	2818983	2843315	24333				
STB-L/STB-G RC1	2	42210	2788188	2802697	14510				
			2811496	2835087	23592				
STB-L/STB-G RC2	6	290158	57511	100987	43477				
			111032	151010	39979				
			2788175	2795837	7663				
			3008248	3116366	108119				
			3283885	3286846	2962				
			3492240	3580197	87958				
STB-L/STB-I RC1	5	53153	1444910	1452225	7316				
			2294501	2306891	12391				
			2317084	2317669	586				
			2335391	2344029	8639				
			2822559	2846779	24221				
STB-D/STB-I RC1	4	84057	2810058	2847942	37885				
			2853406	2865410	12005				
			2865411	2867193	1783	DS	2832659	2838658	6000
			2867194	2899577	32384				
STB-L/STB-K RC1*	2	74209	819417	833644	14228	*donor received the pMRF1-dsred plasmid			
			833645	834625	981	DS	851351	856646	5296
			834626	877259	42634				
			3240460	3256825	16366				
STB-L/STB-K RC2*	0	0	-	-	0	*donor received the pMRF1-dsred plasmid			
STB-G/STB-K RC1*	0	0	-	-	0	*donor received the pMRF1-dsred plasmid			
STB-G/STB-E RC1*	4	60190	2261319	2265252	3934	*donor received the pMRF1-dsred plasmid			
			2267099	2274587	7489				
			2297053	2318975	21923				
			2349463	2376306	26844				
