# Supplementary material for: ESX-1-Independent Horizontal Gene Transfer by Mycobacterium tuberculosis Complex Strains
Source: mBio. 2021 May 18;12(3):e00965-21. doi: 10.1128/mBio.00965-21 (PMC8262963; doi:10.1128/mBio.00965-21)
Supplement: TABLE S2 [file mbio.00965-21-st002.pdf]

Table S2: Mycobacterial strains used in this work

strain	strain code	reference/note
STB-A	CIPT 140010059	(1)
STB-D	CIPT 140060008	(1)
STB-K	CIPT 140070010	(1)
STB-L	CIPT 140070008	(1)
STB-G	CIPT 140070005	(1)
STB-I	CIPT 140070007	(1)
STB-E	CIPT 140070002	(1)
M. tuberculosis EAI	1400-10134	(2)
M. tuberculosis TB36	TB36	(2)
M. tuberculosis Beijing	2002 0554	
M. tuberculosis Delhi	2005 0318	(2)
M. tuberculosis H37Rv		(3)
M. africanum 65	CIPT 140030065	(4)
M. africanum 01	CIPT 140030001	(4)
M. microti	ATCC 35782	(5)
M. orygis	800564	(6)
M. pinnipedii	CIPT 140090001	Pasteur strain collection
M. caprae	CIPT 140080001	Pasteur strain collection
M. bovis	AF2122/97	(7)
M. bovis BCG Russia	BCG-1	(8)
M. bovis BCG Tokyo	172	(9)
M. bovis BCG Pasteur	1173P2	(10)
M. bovis BCG Pasteur 2F9		(11)
M. kansasii	ATCC 12478	(12)
M. lacus	ATCC BAA-323	(13)

References

1. Supply P, Marceau M, Mangenot S, Roche D, Rouanet C, Khanna V, Majlessi L, Criscuolo A, Tap J, Pawlik A, Fiette L, Orgeur M, Fabre M, Parmentier C, Frigui W, Simeone R, Boritsch EC, Debrie A-S, Willery E, Walker D, Quail MA, Ma L, Bouchier C, Salvignol G, Sayes F, Cascioferro A, Seemann T, Barbe V, Locht C, Gutierrez M-C, Leclerc C, Bentley SD, Stinear TP, Brisse S, Médigue C, Parkhill J, Cruveiller S, Brosch R. 2013.
Genomic analysis of smooth tubercle bacilli provides insights into ancestry and pathoadaptation of Mycobacterium tuberculosis. Nature Genetics 45:172-179.

2. Bottai D, Frigui W, Sayes F, Di Luca M, Spadoni D, Pawlik A, Zoppo M, Orgeur M, Khanna V, Hardy D, Mangenot S, Barbe V, Medigue C, Ma L, Bouchier C, Tavanti A, Larrouy-Maumus G, Brosch R. 2020.
TbD1 deletion as a driver of the evolutionary success of modern epidemic Mycobacterium tuberculosis lineages. Nature Communications 11:684.

3. Cole ST, Brosch R, Parkhill J, Garnier T, Churcher C, Harris D, Gordon SV, Eiglmeier K, Gas S, Barry CE, Tekaia F, Badcock K, Basham D, Brown D, Chillingworth T, Connor R, Davies R, Devlin K, Feltwell T, Gentles S, Hamlin N, Holroyd S, Hornsby T, Jagels K, Krogh A, McLean J, Moule S, Murphy L, Oliver K, Osborne J, Quail MA, Rajandream MA, Rogers J, Rutter S, Seeger K, Skelton J, Squares R, Squares S, Sulston JE, Taylor K, Whitehead S, Barrell BG. 1998.
Deciphering the biology of Mycobacterium tuberculosis from the complete genome sequence. Nature 393:537-544.

4. Ates LS, Dippenaar A, Sayes F, Pawlik A, Bouchier C, Ma L, Warren RM, Sougakoff W, Majlessi L, van Heijst JWJ, Brossier F, Brosch R. 2018. Unexpected Genomic and Phenotypic Diversity of Mycobacterium africanum Lineage 5 Affects Drug Resistance, Protein Secretion, and Immunogenicity. Genome Biol Evol 10:1858-1874.

5. Orgeur M, Frigui W, Pawlik A, Clark S, Williams A, Ates LS, Ma L, Bouchier C, Parkhill J, Brodin P, Brosch R. 2021. Pathogenomic analyses of Mycobacterium microti, an ESX-1-deleted member of the Mycobacterium tuberculosis complex causing disease in various hosts. Microb Genom 7.

6. van Ingen J, Rahim Z, Mulder A, Boeree MJ, Simeone R, Brosch R, van Soolingen D. 2012.
Characterization of Mycobacterium orygis as M. tuberculosis complex subspecies. Emerg Infect Dis 18:653-5.

7. Malone KM, Farrell D, Stuber TP, Schubert OT, Aebersold R, Robbe-Austerman S, Gordon SV. 2017. Updated Reference Genome Sequence and Annotation of Mycobacterium bovis AF2122/97. Genome Announc 5.

8. Abdallah AM, Hill-Cawthorne GA, Otto TD, Coll F, Guerra-Assunção JA, Gao G, Naeem R, Ansari H, Malas TB, Adroub SA, Verboom T, Ummels R, Zhang H, Panigrahi AK, McNerney R, Brosch R, Clark TG, Behr MA, Bitter W, Pain A. 2015. Genomic expression catalogue of a global collection of BCG vaccine strains show evidence for highly diverged metabolic and cell-wall adaptations. Sci Rep 5:15443.

9. Seki M, Honda I, Fujita I, Yano I, Yamamoto S, Koyama A. 2009. Whole genome sequence analysis of Mycobacterium bovis bacillus Calmette-Guérin (BCG) Tokyo 172: a comparative study of BCG vaccine substrains. Vaccine 27:1710-6.

10. Brosch R, Gordon SV, Garnier T, Eiglmeier K, Frigui W, Valenti P, Dos Santos S, Duthoy S, Lacroix C, Garcia-Pelayo C, Inwald JK, Golby P, Garcia JN, Hewinson RG, Behr MA, Quail MA, Churcher C, Barrell BG, Parkhill J, Cole ST. 2007. Genome plasticity of BCG and impact on vaccine efficacy. Proc Natl Acad Sci U S A 104:5596-601.

11. Pym AS, Brodin P, Brosch R, Huerre M, Cole ST. 2002. Loss of RD1 contributed to the attenuation of the live tuberculosis vaccines Mycobacterium bovis BCG and Mycobacterium microti. Mol Microbiol 46:709-17.

12. Wang J, McIntosh F, Radomski N, Dewar K, Simeone R, Enninga J, Brosch R, Rocha EP, Veyrier FJ, Behr MA. 2015. Insights on the emergence of Mycobacterium tuberculosis from the analysis of Mycobacterium kansasii. Genome Biol Evol 7:856-70.

13. Turenne C, Chedore P, Wolfe J, Jamieson F, Broukhanski G, May K, Kabani A. 2002. Mycobacterium lacus sp. nov., a novel slowly growing, non-chromogenic clinical isolate. Int J Syst Evol Microbiol 52:2135-2140.
