# Supplementary material for: ESX-1-Independent Horizontal Gene Transfer by Mycobacterium tuberculosis Complex Strains
Source: mBio. 2021 May 18;12(3):e00965-21. doi: 10.1128/mBio.00965-21 (PMC8262963; doi:10.1128/mBio.00965-21)
Supplement: TABLE S3 [file mbio.00965-21-st003.pdf]

Table S3: Primers used in this work

Primer pair	Sequence (5'-3')	Note/reference
AeccUpFw<br>AeccUpRevZeo	GAAGTGCTGCCGGCACCG<br>GAGCCTTTCGTTATTTAAATCGACGTCCAGCCCTCTCG	Amplification of upstream flanking region of STB-A and STB-D *eccD*$_1$
AeccDnFwZeo<br>AeccDnRev	GTTCCACTGAGCGATTTAAATGCCGGATCCGCTGATTGG<br>GCTGGGTGGCCTGATTG	Amplification of downstream flanking region of STB-A, STB-K and STB-L *eccD*$_1$
DeccDnFwZeo<br>DeccDnRev	GTTCCACTGAGCGATTTAAATGCCGGATCGGCTGATTGG<br>TGGGTAGCCTGACTTGGC	Amplification of downstream flanking region of STB-D *eccD*$_1$
KeccUpFw<br>AeccUpRevZeo	GAAGTTCTGCCGGCACCG<br>GAGCCTTTCGTTATTTAAATCGACGTCCAGCCCTCTCG	Amplification of upstream flanking region of STB-K and STB-L *eccD*$_1$
3step-eccD1-F<br>L-3step-eccD1-R	TTTACTAGTGAAGTTCTGCCGGCACGG<br>TTTTCTAGAGCTGGGTGGCCTGACTTG	Amplification of the STB-L AES with *Spe*I/*Xba*I restriction sites.
ZeoFw<br>ZeoRev	ATTTAAATAACGAAAGGCTCAGTC<br>ATTTAAATCGCTCAGTGGAACG	Amplification of the zeocin cassette
HygroFw<br>HygroRev	AGAGCACCAACCCCGTACT<br>TCCGGGAAGACCTCGGAAT	Confirmation of presence of hygromycin cassette; (14)
KanaFw<br>KanaRev	GCGATAATGTCGGGCAATCA<br>GAGGCAGTTCCATAGGATGG	Confirmation of presence of kanamycin cassette; (14)
eccD1checkFw<br>eccD1checkRev	CGAGAGGGCTGGACGTCG<br>CCGCCAATCAGCGGATCC	Verification of *eccD*$_1$ deletion
EsxAFw<br>ZeocheckRev	ATGACAGAGCAGCAGTGGAA<br>GACCACTCGGCGTACAGCTC	Verification of *eccD*$_1$ deletion
Zeo-int<br>EspJRev	GTGACCCTGTTCATCAGC<br>CAACGTTGTGGTTGTTGAGG	Verification of *eccD*$_1$ deletion
RD5esxOFw<br>RD5glySRev	CTCGACGTGACATTCCGAGG<br>GCTATCGGGCGGCGTTAC	Primers flanking the RD5 region
RD5esxOFw<br>BN43_31612Rev	CTCGACGTGACATTCCGAGG<br>GCTGTGCCACCCGATGATTA	Forward primer outside the RD5 region, reverse primer inside RD5
Cas6-bovis-Fw<br>Cas6-bovis-Rev	GATCTGCTACGTCGGAGTGG<br>CTCGCCGGATTGCTTGAATG	Confirmation of presence of *M. bovis cas6* gene
Cas10-bovis-Fw<br>Cas10-bovis-Rev	ACACTCACGTTCTGTAGCCG<br>CCGGAGTAGATGATCGTGGC	Confirmation of presence of *M. bovis cas10* gene
Cas3-stbL-Fw<br>Cas3-stbL-Rev	CGACGTGACCATGTCTTGGA<br>GCTGTCAGTGGTGGATGTGA	Confirmation of presence of STB-L *cas3* gene

14. Boritsch EC, Khanna V, Pawlik A, Honoré N, Navas VH, Ma L, Bouchier C, Seemann T, Supply P, Stinear TP, Brosch R. 2016. Key experimental evidence of chromosomal DNA transfer among selected tuberculosis-causing mycobacteria. Proceedings of the National Academy of Sciences doi:10.1073/pnas.1604921113:201604921.
