# Supplementary material for: ESX-1-Independent Horizontal Gene Transfer by Mycobacterium tuberculosis Complex Strains
Source: mBio. 2021 May 18;12(3):e00965-21. doi: 10.1128/mBio.00965-21 (PMC8262963; doi:10.1128/mBio.00965-21)
Supplement: TABLE S4 [file mbio.00965-21-st004.pdf]

**Table S4**. List of accession numbers for the genome data used or generated in this study.

| Strain | Type | Accesion number | Reference |
|---|---|---|---|
| *Mycobacterium africanum* 01 | Reference genome | NZ_LR993210.1 | 4 |
| *Mycobacterium africanum* 65 | Reference genome | NZ_LR993208.1 | 4 |
| *Mycobacterium bovis* AF2122/97 | Reference genome | LT708304.1 | 7 |
| *Mycobacterium bovis* BCG Pasteur 1173P2 | Reference genome | AM408590.1 | 10 |
| *Mycobacterium bovis* BCG Russia | Reference genome | NZ_CUWO01000001.1 | 8 |
| *Mycobacterium bovis* BCG Tokyo 172 | Reference genome | AP010918.1 | 9 |
| *Mycobacterium canettii* CIPT 140010059 STB-A | Reference genome | HE572590.1 | 1 |
| *Mycobacterium canettii* CIPT 140060008 STB-D | Reference genome | FO203507.1 | 1 |
| *Mycobacterium canettii* CIPT 140070002 STB-E | Reference genome | NZ_LR993206.1 | 1 |
| *Mycobacterium canettii* CIPT 140070005 STB-G | Reference genome | NZ_LR993207.1 | 1 |
| *Mycobacterium canettii* CIPT 140070007 STB-I | Reference genome | NZ_LR993209.1 | 1 |
| *Mycobacterium canettii* CIPT 140070010 STB-K | Reference genome | FO203509.1 | 1 |
| *Mycobacterium canettii* CIPT 140070008 STB-L | Reference genome | FO203508.1 | 1 |
| *Mycobacterium microti* ATCC 35782 | Reference genome | NZ_LR882496.1 | 5 |
| *Mycobacterium tuberculosis* H37Rv | Reference genome | AL123456.3 | 3 |
| *Mycobacterium africanum* 01 donor | Sequencing reads | ERR5102806 | This study |
| *Mycobacterium africanum* 65 donor | Sequencing reads | ERR5102807 | This study |
| *Mycobacterium bovis* AF2122/97 donor | Sequencing reads | ERR5102808 | This study |
| *Mycobacterium bovis* BCG Pasteur donor | Sequencing reads | ERR5102809 | This study |
| *Mycobacterium bovis* BCG Russia donor | Sequencing reads | ERR5102810 | This study |
| *Mycobacterium bovis* BCG Tokyo donor | Sequencing reads | ERR5102811 | This study |
| *Mycobacterium microti* donor | Sequencing reads | ERR5102812 | This study |
| *Mycobacterium tuberculosis* H37Rv donor | Sequencing reads | ERR5102813 | This study |
| *Mycobacterium tuberculosis* H37Rv ΔRD1 donor | Sequencing reads | ERR5102814 | This study |
| STB-A donor | Sequencing reads | ERR5104570 | This study |
| STB-A Δ*eccD1* donor | Sequencing reads | ERR5104571 | This study |
| STB-A ΔRD1 donor | Sequencing reads | ERR5104572 | This study |
| STB-D donor | Sequencing reads | ERR5104573 | This study |
| STB-D Δ*eccD1* donor | Sequencing reads | ERR5104574 | This study |
| STB-G donor | Sequencing reads | ERR5104576 | This study |
| STB-K donor | Sequencing reads | ERR5104577 | This study |
| STB-K Δ*eccD1* donor | Sequencing reads | ERR5104578 | This study |
| STB-L donor | Sequencing reads | ERR5104579 | This study |
| STB-E recipient | Sequencing reads | ERR5106562 | This study |
| STB-G recipient | Sequencing reads | ERR5106563 | This study |
| STB-I recipient | Sequencing reads | ERR5106564 | This study |
| STB-K recipient | Sequencing reads | ERR5106565 | This study |
| STB-L recipient | Sequencing reads | ERR5106566 | This study |
| STB-L Δ*eccD1* recipient | Sequencing reads | ERR5106567 | This study |
| *Mycobacterium africanum* 01/STB-L RC1 | Sequencing reads | ERR5159436 | This study |
| *Mycobacterium africanum* 01/STB-L RC2 | Sequencing reads | ERR5159437 | This study |
| *Mycobacterium africanum* 65/STB-L RC1 | Sequencing reads | ERR5159438 | This study |
| *Mycobacterium africanum* 65/STB-L RC2 | Sequencing reads | ERR5159439 | This study |
| *Mycobacterium bovis*/STB-L RC1 | Sequencing reads | ERR5159440 | This study |
| *Mycobacterium bovis*/STB-L RC2 | Sequencing reads | ERR5159441 | This study |
| *Mycobacterium bovis*/STB-L Δ*eccD1* RC1 | Sequencing reads | ERR5159442 | This study |
| *Mycobacterium bovis* BCG Pasteur/STB-L RC1 | Sequencing reads | ERR5159443 | This study |
| *Mycobacterium bovis* BCG Pasteur/STB-L RC2 | Sequencing reads | ERR5159444 | This study |
| *Mycobacterium bovis* BCG Pasteur/STB-L RC3 | Sequencing reads | ERR5159445 | This study |
| *Mycobacterium bovis* BCG Pasteur/STB-L RC4 | Sequencing reads | ERR5159446 | This study |
| *Mycobacterium bovis* BCG Russia/STB-L RC1 | Sequencing reads | ERR5159447 | This study |
| *Mycobacterium bovis* BCG Tokyo/STB-L RC1 | Sequencing reads | ERR5159448 | This study |
| *Mycobacterium bovis* BCG Tokyo/STB-L RC2 | Sequencing reads | ERR5159449 | This study |
| *Mycobacterium microti*/STB-G RC1 | Sequencing reads | ERR5159450 | This study |
| *Mycobacterium microti*/STB-L RC1 | Sequencing reads | ERR5159451 | This study |
| *Mycobacterium microti*/STB-L RC2 | Sequencing reads | ERR5159452 | This study |
| *Mycobacterium tuberculosis* H37Rv/STB-L RC1 | Sequencing reads | ERR5159453 | This study |
| *Mycobacterium tuberculosis* H37Rv ΔRD1/STB-L RC1 | Sequencing reads | ERR5159454 | This study |

| | | | |
|---|---|---|---|
| STB-A/STB-L RC1 | Sequencing reads | ERR5163979 | This study |
| STB-A Δ*eccD1*/STB-L RC1 | Sequencing reads | ERR5163980 | This study |
| STB-A ΔRD1/STB-L RC1 | Sequencing reads | ERR5163981 | This study |
| STB-A ΔRD1/STB-L RC2 | Sequencing reads | ERR5163982 | This study |
| STB-D/STB-I RC1 | Sequencing reads | ERR5163983 | This study |
| STB-D/STB-L RC1 | Sequencing reads | ERR5163984 | This study |
| STB-D Δ*eccD1*/STB-G RC1 | Sequencing reads | ERR5163985 | This study |
| STB-G/STB-E RC1 | Sequencing reads | ERR5163986 | This study |
| STB-G/STB-K RC1 | Sequencing reads | ERR5163987 | This study |
| STB-G/STB-L RC1 | Sequencing reads | ERR5163988 | This study |
| STB-G/STB-L RC2 | Sequencing reads | ERR5163989 | This study |
| STB-K/STB-G RC1 | Sequencing reads | ERR5165871 | This study |
| STB-K/STB-L RC1 | Sequencing reads | ERR5165872 | This study |
| STB-K Δ*eccD1*/STB-L RC1 | Sequencing reads | ERR5165873 | This study |
| STB-L/STB-G RC1 | Sequencing reads | ERR5165874 | This study |
| STB-L/STB-G RC2 | Sequencing reads | ERR5165875 | This study |
| STB-L/STB-I RC1 | Sequencing reads | ERR5165876 | This study |
| STB-L/STB-K RC1 | Sequencing reads | ERR5165877 | This study |
| STB-L/STB-K RC2 | Sequencing reads | ERR5165878 | This study |

References


1. Supply P, Marceau M, Mangenot S, Roche D, Rouanet C, Khanna V, Majlessi L, Criscuolo A, Tap J, Pawlik A, Fiette L, Orgeur M, Fabre M, Parmentier C, Frigui W, Simeone R, Boritsch EC, Debrie A-S, Willery E, Walker D, Quail MA, Ma L, Bouchier C, Salvignol G, Sayes F, Cascioferro A, Seemann T, Barbe V, Locht C, Gutierrez M-C, Leclerc C, Bentley SD, Stinear TP, Brisse S, Médigue C, Parkhill J, Cruveiller S, Brosch R. 2013.
Genomic analysis of smooth tubercle bacilli provides insights into ancestry and pathoadaptation of Mycobacterium tuberculosis. Nature Genetics
45:172-179.

3. Cole ST, Brosch R, Parkhill J, Garnier T, Churcher C, Harris D, Gordon SV, Eiglmeier K, Gas S, Barry CE, Tekaia F, Badcock K, Basham D, Brown D, Chillingworth T, Connor R, Davies R, Devlin K, Feltwell T, Gentles S, Hamlin N, Holroyd S, Hornsby T, Jagels K, Krogh A, McLean J, Moule S, Murphy L, Oliver K, Osborne J, Quail MA, Rajandream MA, Rogers J, Rutter S, Seeger K, Skelton J, Squares R, Squares S, Sulston JE, Taylor K, Whitehead S, Barrell BG. 1998.
Deciphering the biology of Mycobacterium tuberculosis from the complete genome sequence. Nature 393:537-544.

4. Ates LS, Dippenaar A, Sayes F, Pawlik A, Bouchier C, Ma L, Warren RM, Sougakoff W, Majlessi L, van Heijst JWJ, Brossier F, Brosch R. 2018. Unexpected Genomic and Phenotypic Diversity of Mycobacterium africanum Lineage 5 Affects Drug Resistance, Protein Secretion, and Immunogenicity. Genome Biol Evol 10:1858-1874.

5. Orgeur M, Frigui W, Pawlik A, Clark S, Williams A, Ates LS, Ma L, Bouchier C, Parkhill J, Brodin P, Brosch R. 2021. Pathogenomic analyses of
Mycobacterium microti, an ESX-1-deleted member of the Mycobacterium tuberculosis complex causing disease in various hosts. Microb Genom 7.

7. Malone KM, Farrell D, Stuber TP, Schubert OT, Aebersold R, Robbe-Austerman S, Gordon SV. 2017. Updated Reference Genome Sequence and Annotation of Mycobacterium bovis AF2122/97. Genome Announc 5.

8. Abdallah AM, Hill-Cawthorne GA, Otto TD, Coll F, Guerra-Assunção JA, Gao G, Naeem R, Ansari H, Malas TB, Adroub SA, Verboom T, Ummels R, Zhang H, Panigrahi AK, McNerney R, Brosch R, Clark TG, Behr MA, Bitter W, Pain A. 2015. Genomic expression catalogue of a global collection of BCG vaccine strains show evidence for highly diverged metabolic and cell-wall adaptations. Sci Rep 5:15443.

9. Seki M, Honda I, Fujita I, Yano I, Yamamoto S, Koyama A. 2009. Whole genome sequence analysis of Mycobacterium bovis bacillus Calmette-Guérin (BCG) Tokyo 172: a comparative study of BCG vaccine substrains. Vaccine 27:1710-6.

10. Brosch R, Gordon SV, Garnier T, Eiglmeier K, Frigui W, Valenti P, Dos Santos S, Duthoy S, Lacroix C, Garcia-Pelayo C, Inwald JK, Golby P, Garcia JN, Hewinson RG, Behr MA, Quail MA, Churcher C, Barrell BG, Parkhill J, Cole ST. 2007. Genome plasticity of BCG and impact on vaccine efficacy. Proc Natl Acad Sci U S A 104:5596-601.
